# Broad Anatomical Variation within a Narrow Wood Density Range—A Study of Twig Wood across 69 Australian Angiosperms

**DOI:** 10.1371/journal.pone.0124892

**Published:** 2015-04-23

**Authors:** Kasia Ziemińska, Mark Westoby, Ian J. Wright

**Affiliations:** Department of Biological Sciences, Macquarie University, Sydney, NSW 2109, Australia; INRA—University of Bordeaux, FRANCE

## Abstract

**Objectives:**

Just as people with the same weight can have different body builds, woods with the same wood density can have different anatomies. Here, our aim was to assess the magnitude of anatomical variation within a restricted range of wood density and explore its potential ecological implications.

**Methods:**

Twig wood of 69 angiosperm tree and shrub species was analyzed. Species were selected so that wood density varied within a relatively narrow range (0.38–0.62 g cm^-3^). Anatomical traits quantified included wood tissue fractions (fibres, axial parenchyma, ray parenchyma, vessels, and conduits with maximum lumen diameter below 15 μm), vessel properties, and pith area. To search for potential ecological correlates of anatomical variation the species were sampled across rainfall and temperature contrasts, and several other ecologically-relevant traits were measured (plant height, leaf area to sapwood area ratio, and modulus of elasticity).

**Results:**

Despite the limited range in wood density, substantial anatomical variation was observed. Total parenchyma fraction varied from 0.12 to 0.66 and fibre fraction from 0.20 to 0.74, and these two traits were strongly inversely correlated (*r* = -0.86, *P* < 0.001). Parenchyma was weakly (0.24 *≤*|*r|≤* 0.35, *P* < 0.05) or not associated with vessel properties nor with height, leaf area to sapwood area ratio, and modulus of elasticity (0.24 *≤*|*r|≤* 0.41, *P* < 0.05). However, vessel traits were fairly well correlated with height and leaf area to sapwood area ratio (0.47 *≤*|*r|≤* 0.65, all *P* < 0.001). Modulus of elasticity was mainly driven by fibre wall plus vessel wall fraction rather than by the parenchyma component.

**Conclusions:**

Overall, there seem to be at least three axes of variation in xylem, substantially independent of each other: a wood density spectrum, a fibre-parenchyma spectrum, and a vessel area spectrum. The fibre-parenchyma spectrum does not yet have any clear or convincing ecological interpretation.

## Introduction

There are many ways plants make a living, or many ecological ‘strategies’, and the study of plant traits—and their variation among species—is a key way of gaining insight into these [1–3]. Some strategies tend to be related to wood density (g cm^-3^; [4]), but plausibly there are also strategies related to wood properties that are independent of density. Wood density is one of the most commonly measured ecological traits, but its direct functional meaning is not entirely clear. One reason might be that wood density is just one value describing a complex structure with multiple functions. On the basis that density is directly determined by anatomical structure, several studies have searched for links between density and anatomy [5–13]. Variation in wood density across species has proved to be mainly driven by fibre wall and fibre lumen properties (e.g. fibre wall and lumen fractions), more so than by the fraction of wood that is vessels [5–8,12–14], creating one dimension of ecological trait variation.

At the same time, species with similar wood densities might actually have diverse anatomies [12]. Little is known about the magnitude of this sort of variation, and how it might relate to potentially unexplored dimensions of ecological strategy. The present work selects species within a fairly narrow density range (0.38–0.62 g cm^-3^, 1.6-fold variation compared to worldwide variation of *c*. 17-fold, [15]). The 0.38–0.62 density range encompasses values studied previously [12], but in that study they were represented by a few species only. Here we aim to look at a wider set of species to better quantify anatomical variation within this restricted range of wood density.

Angiosperm wood is composed of several distinct tissues or cell types: fibres, parenchyma, and vessels. Their main functions are considered to be, respectively, mechanical support for fibres, metabolite transport and storage and water storage for parenchyma, and water transport via vessels [16]. Among these tissues, parenchyma appears to be functionally most diverse yet its ecological and physiological role on a broad, interspecific scale remains poorly understood. It has been suggested that parenchyma may play several hydraulic roles, for example, water storage, capacitance [17] and vessel refilling after embolism [18–25]. The question whether parenchyma contributes to embolism refilling is the topic of an ongoing debate with evidence both for [18–25] and against[26–30]. Studying relationships between vessel size (the smaller the vessel the less prone to embolism) and parenchyma traits in the context of different environmental conditions may contribute to the discussion on xylem hydraulic functions. Other parenchyma roles include pathogen defence, wound closure, tylosis formation and storage of mineral components [16,31–36]. Parenchyma may also contribute to wood mechanical performance. For instance, ray parenchyma which stretches radially from pith to cambium has been shown to increase with modulus of elasticity, though that study encompassed only five species [37].

Most studies, together spanning more than a thousand species, report that parenchyma fraction and fibre fraction vary independently from wood density or with only weak correlation, but that parenchyma and fibre are strongly negatively correlated with each other [8–13]. Because parenchyma and fibres are the two most abundant tissues, there is therefore a dimension of anatomical variation running from high parenchyma fraction to high fibre fraction (but with a good share of the fibre being lumen in lower-density species); this dimension is substantially independent from wood density variation [12]. Previous work on twigs showed that this dimension was widest in medium-density species (roughly 0.50–0.75 g cm^-3^ in a study spanning 0.38–0.83 g cm^-3^ [12]). However, in that sample only three species had low density (roughly <0.5 g cm^-3^), and logically a wide variation in fibre and parenchyma fractions should be possible in low-density wood.

The present study addressed two broad groups of questions: 1) What is the nature and scope of anatomical variation in low- to medium-density woods (0.38–0.62 g cm^-3^) across a broad range of angiosperm species? 2) What can be inferred about the ecological or functional meaning of this variation? In answering these questions we also aimed to gauge how much of the trait variation between species took the form of variation across different climates, versus how much occurred as variation among coexisting species.

## Materials and Methods

### Plant material and sites

Three sites were chosen along the east coast of Australia, in New South Wales (NSW) and Queensland (QLD), in such a way as to give rise to contrasts both of temperature and of precipitation (site details in Table 1, site photographs in S1 Fig). A cool temperate forest site was located in Kosciuszko National Park, NSW (36.48°S). The two warm sites were located in Queensland: a tropical woodland in Girringun National Park (18.16°S) and a tropical wet rainforest in Daintree National Park (16.10°S). The two tropical sites have similar mean annual temperature of *c*. 22°C but vary markedly in mean annual precipitation (4230 mm and 995 mm respectively), with the lower-rainfall woodland also having a more severe dry season around three times longer (i.e., 9 months *vs*. 3 months; Table 1). Mean annual temperature at the temperate site (*c*. 7°C, being at 1300m elevation) is approximately 15°C lower than at the tropical sites, and mean annual precipitation is 1835 mm of rainfall or equivalent snow. A canopy crane at the Daintree Rainforest Observatory (James Cook University) was used to collect twigs from tall trees. Shorter species were collected from the ground, and all species were collected within one kilometre radius from the crane. Species in the tropical woodland and the temperate forest were collected within a radius of *c*. 5 kilometres from the coordinates provided in Table 1 (except for two species in the tropical woodland, *Lophostemon suaveolens*, *c*. 10 km north west, and *Grevillea glauca*, *c*. 30 km north west). These sites were less rich in species, hence the wider search radius. Queensland Department of Environment and Resource Management issued a permit for work in Daintree and Girringun National Parks (permit WITK09188111). Several species near the border with Girringun National Park were collected on private land with permission from the owners Ross and Maxine Blennerhasset. New South Wales Office of Environment and Heritage issued a permit for work in Kosciuszko National Park (scientific license SL100517).

**Table 1:** Site details.

	Cape Tribulation, Daintree NP	Blencoe Falls, Girringun NP	Thredbo, Kosciuszko NP
Vegetation	Tropical rainforest	Tropical woodland	Temperate forest
Coordinates	16.104°S	18.162°S	36.482°S
	145.449°E	145.490°E	148.350°E
Altitude (m)	50	650	1300
Sampling time	2012 May	2012 Oct	2012 Mar
Mean annual temperature (°C)	22.2	22.4	7.2
Maximum temperature of the warmest month (°C)	29.1	31.2	20.5
	Dec, Jan	Dec	Jan
Maximum temperature of the coldest month (°C)	14.8	11.6	−1.6
	Jul	Jul	Jul
Annual precipitation (mm)	4229	995	1834
Precipitation of the wettest month (mm)	817	197	210
	Mar	Feb	Oct
Precipitation of the driest month (mm)	71	18	86
	Oct	Sep	Feb
Number of months with precipitation below 100mm	3	9	1
	Aug-Oct	Apr-Dec	Feb
Number of months with minimum temperature below 0°C	0	0	5
			May-Sep

Notes: Temperature based on gridded 5km resolution data for 1961–2007. Precipitation data collected at the local weather stations (Cape Tribulation Store, Goshen and Thredbo Village stations) within 5 km from the sites for 1971–1990. All data obtained from Australian Bureau of Meteorology. NP—national park.

Twigs of more than 100 species of trees and shrubs were collected in total, from three replicate individuals per species with a few exceptions stated below. None of the species were protected. Species in the rainforest were targeted using a dataset of twig wood density as a guide (dataset compiled by S.A. Stuart, unpublished). From tropical woodland and temperate forest approximately 30 of the most abundant species were collected, wood density was measured and species with mean density ranging from 0.38 to 0.62 g cm^-3^ were selected for further analysis. The lower density boundary was equal to the lowest-density measured across species sampled here. The higher cut-off was set so that each site was represented by at least ten species. This selection process resulted in the total of 68 species described here, spanning 48 genera and 26 families (S1 Table). One species, *Pimelea linifolia*, occurred in both the tropical woodland and the temperate forest. However, it was measured separately (three replicates per species per each site) and hence treated as two separate entities. Consequently, all the analyses were run on 69 species-at-sites, which hereafter are referred to as ‘69 species’. Three individuals per species were sampled with the following exceptions: four individuals for *Dysoxylum parasiticum* and *D*. *pettigrewianum*, two individuals for *Palaquium galactoxylum*, one individual for *Pouteria xerocarpa* and *Grevillea glauca*. All species are evergreen except four deciduous trees from the rainforest site (*Dysoxylum pettigrewianum*, *Ficus variegata*, *Palaquium galactoxylum* and *Wrightia laevis*). Two rainforest species are pioneers (*Leea indica* and *Mallotus paniculatus*) and one species from the temperate woodland (*Exocarpos strictus*) is a hemi-parasitic shrub, parasitizing roots of neighbouring trees, at least in the early phases of life.

Upper branches, one per individual plant, were collected and processed within 24 hours. A branch of approximately one meter length was divided into four main adjacent sections, using a wood diameter of *c*. 0.5 cm (under bark and excluding pith, i.e. the radius stretched from the outside of the pith to cambium) as a reference point. From the top of the branch the adjacent sections were as follows: 1) the segment above wood diameter of *c*. 0.5 cm for measurements of leaf area to sapwood area ratio, 2) a segment *c*. 10 cm long for anatomical measurements, 3) a segment *c*. 5 cm long for wood density measurement and 4) the remaining segment for mechanical tests detailed below. Occasionally, it was impossible to collect upper branches from tall canopy species (*Eucalyptus* and *Corymbia* species in the temperate forest and the tropical woodland). In those instances, shorter trees or lower branches were sampled.

### Height

The height of each individual tree and shrub sampled was measured using a measuring tape dropped from the crane gondola for tall species (at the rainforest site) or a measuring tape and a clinometer for shorter species at the rainforest site and for all species at the tropical woodland and the temperate forest sites. Additionally, maximum heights of species were recorded from Flora of Australia (*Flora of Australia Online* 2013), from taxonomic literature, or in the absence of those, from online reports.

### Wood density

Within 24 hours from collection, bark and pith were removed and wood was soaked in water for 48 hours. Wood volume was then measured using the buoyancy principle of Archimedes. A container filled with water was placed on a precision balance and a thin wire platform was suspended in the water so that it did not touch any walls or the bottom of the container. A twig was delicately placed on the wire platform and the mass of displaced water was recorded. The balance was tared before each measurement. The mass of displaced water was then used to calculate the volume of a twig assuming standard water density of 1.0 g cm^-3^. Next, twigs were dried for at least 72 hours at 105°C and mass was measured on a precision balance. The density was calculated as dry mass divided by the volume of soaked wood (g cm^-3^).

### Mechanical traits

Mechanical measurements were carried out within two weeks of collection (the number of replicates was as stated above except for two replicates of *Pimelea linifolia* from the temperate forest, one replicate of *P*. *linifolia* from the tropical woodland and two replicates for *Syzygium graveolens* from the tropical rainforest). Between collection and testing, twigs were stored in sealed plastic bags in a cool room where available (4°C) or in an air conditioned room (around 20°C). A material testing machine (Model 5542, Instron Corporation, Canton, MA, USA) was used to carry out a three-point bending test. The segments used to measure modulus of elasticity (MOE) were at least 20 times longer than diameter (including bark and pith), as in previous studies on twigs (e.g. [38,39]). The 1:20 ratio of segment length to the diameter aimed to minimize the effect of shear stress on the compression and tensile stresses, the two principal components of modulus of elasticity [40].

### Anatomical traits

**Fig 1:**
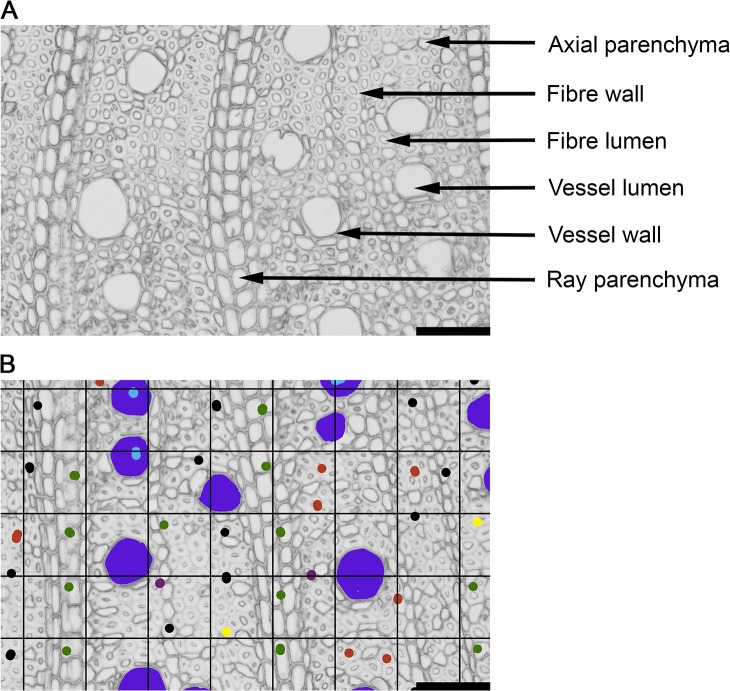
Illustration of wood tissues (A) and the image analysis method applied in this study (B), shown on an example of *Gomphandra australiana*, Icacinaceae. (B) Vessels were manually coloured (large circles in dark blue) and then the vessel areas ware measured in image analysis software (see text for details). Grid method was used to estimate tissue fractions. Grid points were marked according to the tissue they fell in: light blue—vessel lumen, purple—vessel wall, red—axial parenchyma, green—ray parenchyma, yellow—fibre lumen and black—fibre wall. For clarity, the picture illustrates only a fragment of a larger, pie-shape area analysed. Scale bar corresponds to 100 μm.

The anatomical traits measured were average cross-sectional area of vessel lumens (called here ‘vessel area’), pith area, and the total fractions of cross-section contributed by each of vessel lumen, vessel wall, axial parenchyma, ray parenchyma (called ‘rays’ for short), fibre wall, fibre lumen, conduits with maximum lumen diameter below 15 μm (see below), and mucilage canals (Fig 1A). Anatomical definitions followed ‘IAWA list of microscopic features for hardwood identification’ (IAWA Committee 1989). Traditionally angiosperm wood is referred to as a complex tissue composed of various cell types [16]. Nevertheless, the various cell types are called here ‘tissues’ for brevity and because they differ in morphology and functions. In addition to vessels, we established a category of conduits with maximum lumen diameter below 15 μm (called hereafter conduits_15μm_). These were cells with overall diameter in between or similar to that of small vessels and fibres. The walls resembled those of vessels in thickness, pit diameter, and distance between pits on circumference of the cell wall [36]. These cells would have been either tracheids or small vessels, however we were unable to confidently categorize them as one or the other from the cross-sections, hence the category conduits_15μm_. In one species, *Pimelea linifolia*, cells that could have been either thin-walled fibres or axial parenchyma were observed. Those cells formed irregular patterns from a variable thickness band occurring off to one side of the pith to composing an entire growth ring. This morphology resembled that of a ground tissue. Wall thickness varied from that of fibres to that of axial parenchyma. Their diameters ranged from similar to fibres to as large as vessels. Nuclei and starch were not observed in these cells; pits and transverse walls were found only in a couple of cells. The transition between fibres and these cells within a growth ring was gradual and no clear cut-off was observed in wall thickness or cell diameter. However, a clearer difference between axial parenchyma cells associated with vessels and the problematic cells was noted, as the axial parenchyma had abundant simple pits and the problematic cells did not. Based on those observations, the problematic cells were counted as fibres. These cells might be an example of fibre dimorphism where parenchyma-like fibres or parenchyma could have originated from libriform fibres (fibres with small, slit-like pits) and the two cell types are similar in appearance [41].

The twigs were placed in FAA fixative (formalin: acetic acid: 70% ethanol in proportions 5: 5: 90; [42]) within 24 hours after collection. After four weeks the fixative was replaced with 70% ethanol. The ethanol solution was replaced twice more through the following week with the third change of ethanol serving as a storing medium. Before sectioning, samples were placed in 50% ethanol and kept in an oven for up to 72 hours at 40–50°C. This treatment helped to soften the wood. Cross-sections 10–50 μm thick were cut using a sledge microtome (Reichert, Vienna, Austria) and disposable blades (model A35, Feather Safety Razor Co. Ltd, Japan). The sections were mounted in glycerol. High resolution photographs were taken at total 100x magnification using a Nikon digital camera (model DXM 1200F, Nikon Corporation, Japan) mounted on a light microscope (Olympus BX 50F, Olympus Co. Ltd., Japan) and Nikon ACT-1 imaging software (version 2.62, Nikon Corporation, Japan). Each image had dimensions 3840 x 3072 pixels and was saved in tif format. One to two of the most representative radial sectors were chosen and photographed in a sequence from pith to bark. The sectors avoided tension wood where possible. Where the focus was uneven within a field of view, several photographs were taken at different focal lengths. These photographs were then stacked together in Photoshop CS4 (Adobe Systems Incorporated, USA). Next, the photographs capturing the whole radial sector were merged as a sequence resulting in one image of the whole sector. The sector analysed was bounded by pith, rays and bark.

Vessel lumens were coloured in Photoshop and then measured in Image-Pro Plus version 2.0.0.260 (Media Cybernetics Inc., USA). Approximately 30 to 500 vessels per replicate falling within a radial sector were measured. Protoxylem and newly produced vessels were excluded from measurements. To measure tissue fractions a grid of points 300 pixels (84.3 μm) apart in horizontal and vertical directions was overlaid over the image of the radial sector (Fig 1B). A minimum 300 points for each sample were analysed. Using Photoshop, each point was colour coded according to the tissue it fell into. Then image analysis software was used to count points, and to estimate tissue fractions (number of points of a given tissue divided by the total number of analysed points). The grid method has been used previously and is extensively discussed by Smith 1967 [43]; its modified version is applied here. Digital calipers were used to measure two perpendicular diameters of pith on freshly collected twigs, and then the area was calculated as an ellipse.

Additional anatomical traits were calculated as follows. Total parenchyma fraction was the sum of axial and ray parenchyma (called here ‘total parenchyma’). Total fibre fraction was the sum of fibre lumen and fibre wall fractions (called here ‘total fibre’). The sum of fibre wall fraction and vessel wall fraction is called ‘wall_F+V_ fraction’. Also calculated were hydraulically weighted vessel diameter (D_H_), number of vessels per area (N), and the average vessel area to number of vessels per area ratio (S; also called ‘vessel area to number ratio’). D_H_ was calculated from the mean diameters for individual vessels as D_H_ = (Σdiameter^5^)/ (Σdiameter^4^) [44]. N (mm^-2^) was calculated as vessel lumen fraction in the cross-section divided by arithmetic average of vessel lumen area (A, mm^2^). S was calculated as A divided by N (mm^4^; [14].

### Leaf traits

Leaf traits were measured on leaves from twigs with wood diameter of *c*. 0.5 cm (wood diameter measured under bark and excluding pith). For species with compound leaves, the rachis was included in measurements. For each replicate specific leaf area (SLA) was measured on at least seven simple leaves or one to two compound leaves. The number of replicates was as stated above except for two replicates for *Xylomelum scottianum* and *Grevillea parallela* from the tropical woodland.

The leaves were placed in a sealed plastic bag within 24 hours from collection, and stored in a refrigerator for up to a week and a half. Next, they were positioned under transparent plexiglass and photographed (Pentax K100 DSuper, Pentax Ricoh Imaging Company, Japan). The photographs were enhanced in Photoshop and the total area of leaves was measured in Image-Pro Plus. After the photographs were taken the leaves were placed in paper bags and dried in a drying oven at 70°C for at least 72 hours. Leaf dry mass was then measured on a precision balance. SLA was calculated as mass of dried leaves divided by their leaf area.

Leaf area to sapwood area ratio (LA/SA) was calculated for twigs with approximately 0.5 cm of wood diameter (under bark and excluding pith). Two wood diameters perpendicular to each other were measured using digital calipers and sapwood area was calculated as an ellipse. The pith and bark were excluded from the measurement. All leaves from this segment, except for the ones used for SLA, were placed in paper bags and dried at 70°C for at least 72 hours. Next, mass was measured on a precision balance, and the total leaf area on the shoot was calculated as SLA multiplied by the mass of all leaves. The total leaf area at wood diameter of 0.5cm was the sum of the area used for SLA and the remaining leaves. LA/SA was calculated by dividing the total area of leaves by the sapwood cross-sectional area calculated from the two diameters.

### Statistical analysis

Sixty-nine species were analysed, three replicates per species (except for particular cases mentioned above) and species arithmetic trait averages were used in the analyses. Eight out of 22 studied traits showed approximately normal distributions across species (Shapiro-Wilk test, p < 0.05): vessel lumen fraction, ray fraction, fibre lumen fraction, fibre wall fraction, total vessel fraction (vessel lumen + wall), total parenchyma fraction (axial + ray parenchyma), total fibre fraction (fibre lumen + wall), wall_F+V_ (fibre wall + vessel wall) fraction. The remaining traits did not exhibit normal distribution across species: height and maximum height, pith area, density, LA/SA, modulus of elasticity (MOE), vessel wall fraction, axial parenchyma fraction, conduits_15μm_ fraction, mucilage canals fraction, vessel area (A), vessel area to number ratio (S), vessel number per area (N) and hydraulically weighted diameter (D_H_). Among those traits, log-transformations normalized the distributions of three traits only: vessel wall fraction, N and MOE. Consequently, we did not use transformed values but rather ran non-parametric tests. To quantify correlations among traits we used Pearson’s correlation r (for normally distributed variables) and Spearman’s rank correlation coefficient ρ (if one or both variables were not normally distributed). Correlations are described as “significant” when *P* < 0.05, but since a substantial number of correlations are being examined, individual significance tests should not be over-interpreted.

## Results

### Overview of traits

Our first main question was about the scope of anatomical variation in species within a restricted density range (0.38–0.62 g cm^-3^, 1.6 fold variation). First, we report on this topic as well as on variation in height, modulus of elasticity, and leaf area to sapwood area ratio (Table 2).

**Table 2:** Traits overview.

			Range			
Trait	Abbrev.	Units	Low	High	Average	n-fold variation
Axial parenchyma fraction	−	unitless	0.01	0.33	0.14	26.3
Ray parenchyma fraction	ray fraction	"	0.06	0.41	0.21	6.8
Total parenchyma fraction (axial + ray)	–	"	0.12	0.66	0.35	5.7
Fibre lumen fraction	−	"	0.02	0.32	0.13	16.2
Fibre wall fraction	−	"	0.15	0.52	0.32	3.5
Total fibre fraction (lumen + wall)	–	"	0.20	0.74	0.45	3.7
Vessel lumen fraction	−	"	0.06	0.23	0.13	3.9
Vessel wall fraction	−	"	0.02	0.09	0.04	5.4
Total vessel fraction (lumen + wall)	–	"	0.09	0.30	0.18	3.4
Fraction of conduits with maximum lumen diameter smaller than 15 μm	conduits_15μm_ fraction	"	0.002*	0.15	0.06	63.4
Mucilage canals fraction	−	"	0.01*	0.02	0.014	2.2
Wall fraction (fibre wall + vessel wall)	wall_F+V_ fraction	"	0.18	0.56	0.36	3.1
Vessel area	A	mm^2^	0.0002	0.0036	0.0014	16.5
Vessel number per area	N	mm^-2^	34	730.7	148.7	21.5
Vessel area to number ratio	S	mm^4^	0.0000004	0.00011	0.00002	257.7
Hydraulically weighted diameter	D_H_	mm	0.02	0.08	0.05	4.4
Pith area	–	mm^2^	0.13*	72.6	5.6	547.8
Wood density	−	g cm^-3^	0.38	0.62	0.53	1.6
Height	−	m	0.7	33.6	12.2	48.0
Maximum height	−	m	1	40	18.1	40
Modulus of elasticity	MOE	MPa	1555	11778	5200	7.6
Leaf area/sapwood area	–	cm^2^ cm^-2^	838	24904	7884	29.7

Notes: *—this trait's minimum value was 0, the value reported here is the lowest value different from 0, which was used to calculate n-fold variation and mean values.

Among anatomical traits the most variable were pith area (nearly 550-fold variation), and vessel area to number ratio S (>250-fold variation), followed by the conduits_15μm_ fraction (63-fold variation), and the axial parenchyma fraction (26-fold variation). Among non-anatomical traits, the most variable were plant height, with 48-fold variation, and leaf area to sapwood area ratio, with almost 30-fold variation. The least variable anatomical traits were fibre wall fraction, total fibre fraction, total vessel fraction, and mucilage canals fraction, all with approximately 3.5-fold or less variation. The narrow variations in density (1.6-fold) and in fibre wall fraction were to be expected because for this study species were specifically chosen within a relatively narrow band of densities (0.38–0.62 g cm^-3^).

**Fig 2:**
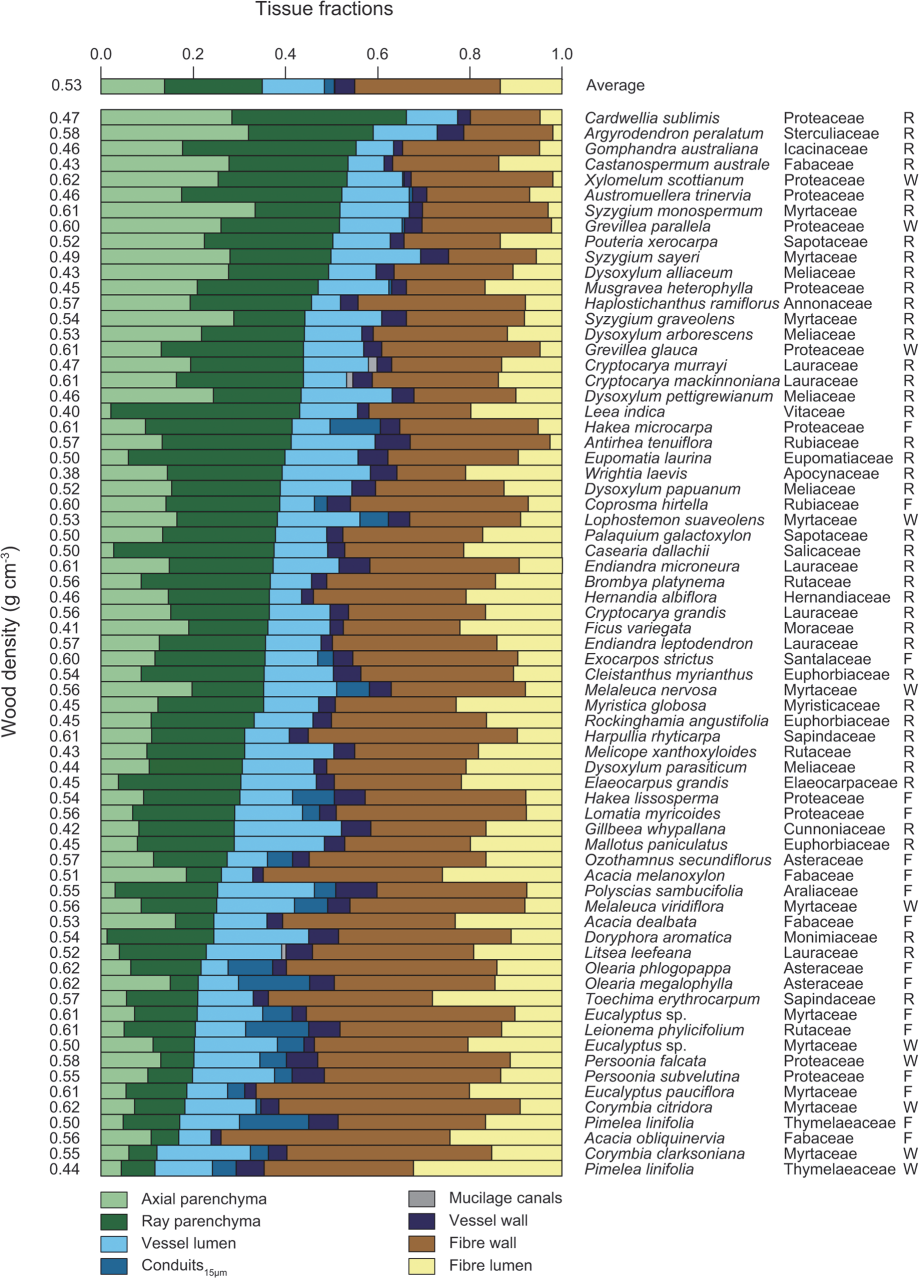
Stack bar graph of tissue fractions across 69 species. Each bar represents an individual species with the top bar representing fractions averaged across all species. Species are sorted in order of decreasing total parenchyma fraction (axial + ray). Numbers on the left side indicate wood density of a given species. Species name, family, and site are given on the right side of the graph. Site codes stand for: R—tropical rainforest (Cape Tribulation), W—tropical woodland (Blencoe Falls) and F—temperate forest (Thredbo).

Overall, there was considerable variation in wood anatomical traits across species (Fig 2). On average, fibres (fibre wall + fibre lumen) were the most abundant tissue with mean fraction of 0.45 and 3.7-fold variation (from 0.20 to 0.74). Fibre wall fraction averaged at 0.32 (with 3.5-fold variation), and fibre lumen fraction averaged at 0.13 (roughly 16-fold variation). The second most abundant tissue was total parenchyma (axial + ray) with mean fraction of 0.35 and almost 6-fold variation (from 0.12 to 0.66). Fig 2 is ordered from highest total parenchyma fraction to lowest (sum of light-green bars, axial parenchyma, and dark-green bars, rays). Parenchyma components, axial and ray, occupied 0.14 (26.3-fold variation) and 0.21 (6.8-fold variation) respectively. Vessels (lumen + wall) occupied on average 0.18 of wood cross-section, and varied 3.4-fold (from 0.09 to 0.30). Their lumen fraction averaged 0.13 with almost 4-fold variation, and wall fraction averaged 0.04 with more than 5-fold variation. Conduits_15μm_ occurred in 26 species (38% of all sampled species) and occupied 0.06 averaged across the 26 species with the conduits_15μm_ present with more than 60-fold variation (or 0.02 averaged across all species). Mucilage canals occurred only in three Lauraceae species (*Cryptocarya murrayi*, *C*. *mackinnoniana*, and *Litsea leefeana*), and they occupied an average 0.01 of the cross-section across those species. This canal fraction was not included in subsequent analysis.

### Anatomical trait relationships

**Fig 3:**
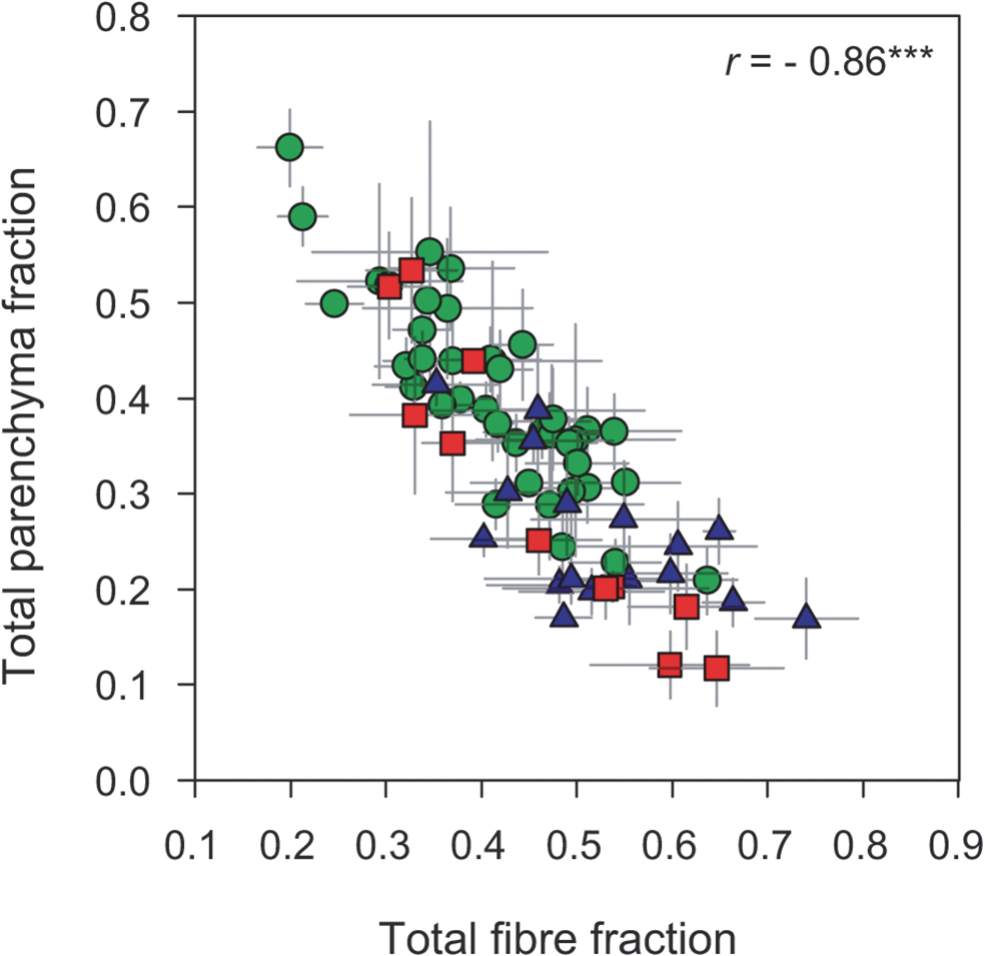
Relationship between total fibre fraction and total parenchyma fraction. Green circles—tropical rainforest (warm and wet site); red squares—tropical woodland (warm and dry site); blue triangles—temperate forest (cool and wet site). *** *P* < 0.001.

Total fibre fraction was strongly negatively correlated with total parenchyma fraction across all species (*r* = - 0.86, *P* < 0.001, Fig 3) as well as within individual sites (data for the individual sites not shown). Fibre wall fraction and fibre lumen fraction did not correlate with each other (*r* = 0.11, *P* = 0.38, but see below for more detail) across all species or within sites. Axial and ray parenchyma were not associated with each other across all species (*ρ* = 0.19, *P* = 0.11) but were positively correlated among the eleven species from the tropical woodland (*ρ* = 0.71, *P* < 0.05). Trait correlation coefficients across all measured traits (except for mucilage canals) are listed in Table 3.

**Table 3:** Pairwise correlations among the studied traits.

		APF	RPF	TPF	FLF	FWF	TFF	VLF	VWF	TVF	CF	WF	A	N	S	Dh	PA	WD	H	MH	MOE	LASA
Axial parenchyma fraction	APF		0.19	**0.72**	**-0.38**	**-0.48**	**-0.61**	-0.13	***-0*.*25***	-0.19	***-0*.*28***	**-0.53**	**0.31**	**-0.35**	**0.36**	***0*.*30***	0.12	-0.02	**0.35**	**0.36**	**-0.34**	0.09
Ray parenchyma fraction*	RPF	0.16		**0.77**	**-0.39**	**-0.52**	**-0.64**	-0.08	-0.12	-0.13	**-0.40**	**-0.54**	0.15	-0.16	0.14	0.09	**0.35**	-0.17	***0*.*27***	0.05	***-0*.*24***	**0.33**
Total parenchyma fraction*	TPF	**0.75**	**0.77**		**-0.52**	**-0.70**	**-0.85**	-0.10	-0.23	-0.17	**-0.46**	**-0.74**	***0*.*30***	***-0*.*29***	***0*.*30***	0.24	**0.35**	-0.16	**0.41**	***0*.*25***	**-0.41**	**0.34**
Fibre lumen fraction*	FLF	**-0.44**	**-0.39**	**-0.55**		0.09	**0.66**	-0.11	***-0*.*24***	-0.14	-0.18	0.03	0.21	***-0*.*28***	***0*.*26***	0.16	0.22	**-0.44**	0.03	0.11	0.14	0.08
Fibre wall fraction*	FWF	**-0.51**	**-0.58**	**-0.72**	0.11		**0.77**	***-0*.*29***	0.01	***-0*.*24***	**0.40**	**0.98**	**-0.46**	0.22	**-0.32**	**-0.34**	**-0.47**	**0.59**	**-0.52**	**-0.36**	**0.47**	**-0.36**
Total fibre fraction*	TFF	**-0.64**	**-0.66**	**-0.86**	**0.69**	**0.79**		***-0*.*25***	-0.13	***-0*.*24***	0.19	**0.72**	-0.16	-0.03	-0.06	-0.10	-0.19	0.14	**-0.33**	-0.18	**0.38**	-0.15
Vessel lumen fraction*	VLF	-0.11	-0.06	-0.11	-0.10	***-0*.*28***	***-0*.*27***		**0.48**	**0.95**	-0.03	-0.19	0.19	***0*.*26***	-0.09	0.19	0.06	***-0*.*27***	**0.31**	***0*.*31***	***-0*.*25***	***0*.*24***
Vessel wall fraction	VWF	***-0*.*25***	-0.13	***-0*.*25***	-0.22	-0.03	-0.16	**0.50**		**0.70**	*0*.*22*	0.19	**-0.42**	**0.66**	**-0.58**	**-0.43**	-0.18	0.10	-0.06	-0.15	0.10	-0.22
Total vessel fraction*	TVF	-0.17	-0.09	-0.17	-0.15	***-0*.*24***	***-0*.*27***	**0.96**	**0.72**		0.06	-0.10	0.02	**0.43**	***-0*.*26***	0.01	-0.02	-0.22	0.23	0.20	-0.17	0.11
Conduits_15μm_ fraction	CF	***-0*.*31***	**-0.41**	**-0.47**	-0.06	***0*.*30***	0.18	-0.14	***0*.*29***	-0.02		**0.45**	**-0.49**	**0.53**	**-0.54**	**-0.38**	**-0.54**	**0.40**	**-0.66**	**-0.52**	0.16	**-0.67**
Wall_F+V_ fraction*	WF	**-0.55**	**-0.59**	**-0.76**	0.06	**0.98**	**0.75**	-0.18	0.16	-0.09	**0.35**		**-0.53**	**0.34**	**-0.43**	**-0.42**	**-0.49**	**0.60**	**-0.53**	**-0.39**	**0.50**	**-0.40**
Vessel area	A	***0*.*24***	0.19	***0*.*28***	0.23	**-0.37**	-0.13	0.17	**-0.45**	-0.00	**-0.53**	**-0.46**		**-0.85**	**0.93**	**0.94**	**0.45**	**-0.47**	**0.64**	**0.65**	**-0.44**	**0.58**
Vessel number per area	N	**-0.38**	-0.16	**-0.35**	-0.18	0.16	0.01	0.18	**0.66**	**0.35**	**0.62**	***0*.*29***	**-0.71**		**-0.97**	**-0.82**	**-0.42**	**0.31**	**-0.47**	**-0.50**	***0*.*26***	**-0.48**
Vessel area to number ratio	S	0.22	0.19	***0*.*27***	***0*.*26***	***-0*.*28***	-0.04	-0.13	**-0.51**	***-0*.*27***	**-0.39**	**-0.37**	**0.88**	**-0.56**		**0.88**	**0.43**	**-0.40**	**0.55**	**0.58**	**-0.31**	**0.52**
Hydr. weighted diameter	Dh	***0*.*31***	0.11	***0*.*27***	0.16	***-0*.*28***	-0.11	0.19	**-0.48**	0.00	**-0.57**	**-0.37**	**0.94**	**-0.79**	**0.76**		**0.35**	**-0.35**	**0.52**	**0.57**	**-0.44**	**0.54**
Pith area	PA	0.01	**0.42**	***0*.*29***	0.02	**-0.34**	-0.23	0.05	-0.19	-0.02	-0.22	**-0.37**	**0.39**	-0.23	**0.38**	***0*.*30***		**-0.52**	**0.48**	0.23	-0.17	**0.66**
Wood density	WD	-0.04	***-0*.*24***	-0.18	**-0.44**	**0.60**	0.17	***-0*.*28***	0.11	-0.19	**0.34**	**0.62**	**-0.54**	***0*.*24***	**-0.51**	**-0.41**	**-0.41**		**-0.36**	**-0.31**	***0*.*27***	**-0.39**
Height	H	**0.38**	***0*.*26***	**0.42**	-0.03	**-0.52**	**-0.40**	***0*.*29***	0.01	0.24	**-0.51**	**-0.51**	**0.48**	**-0.40**	**0.34**	**0.45**	0.15	**-0.36**		**0.83**	**-0.47**	**0.58**
Maximum height	MH	**0.37**	0.02	***0*.*25***	0.09	**-0.32**	-0.18	***0*.*28***	-0.16	0.17	**-0.53**	**-0.34**	**0.53**	**-0.50**	**0.37**	**0.58**	0.02	**-0.33**	**0.78**		**-0.40**	**0.46**
Modulus of elasticity	MOE	**-0.31**	**-0.34**	**-0.43**	0.16	**0.45**	**0.43**	***-0*.*28***	0.15	-0.17	**0.36**	**0.47**	**-0.41**	**0.39**	-0.23	**-0.46**	-0.13	***0*.*29***	**-0.50**	**-0.35**		-0.22
Leaf area/sapwood area	LASA	0.03	**0.39**	***0*.*28***	0.08	***-0*.*29***	-0.16	0.20	-0.23	0.09	**-0.55**	**-0.33**	**0.60**	**-0.46**	**0.48**	**0.55**	**0.59**	**-0.43**	**0.35**	***0*.*29***	***-0*.*27***	

Notes: Trait names abbreviations are derived from first letters of full trait names, except for A—vessel area, N—vessel number per area, S—vessel area to number ratio, and Dh—hydraulically weighted diameter. Significance levels: bold italic—*P* < 0.05, bold—*P* < 0.01, bold underlined—*P* < 0.001. Above diagonal—Spearman correlation coefficients, below diagonal—Pearson correlation coefficients.

*—normally distributed trait values (Shapiro-Wilk test, p < 0.05).

**Fig 4:**
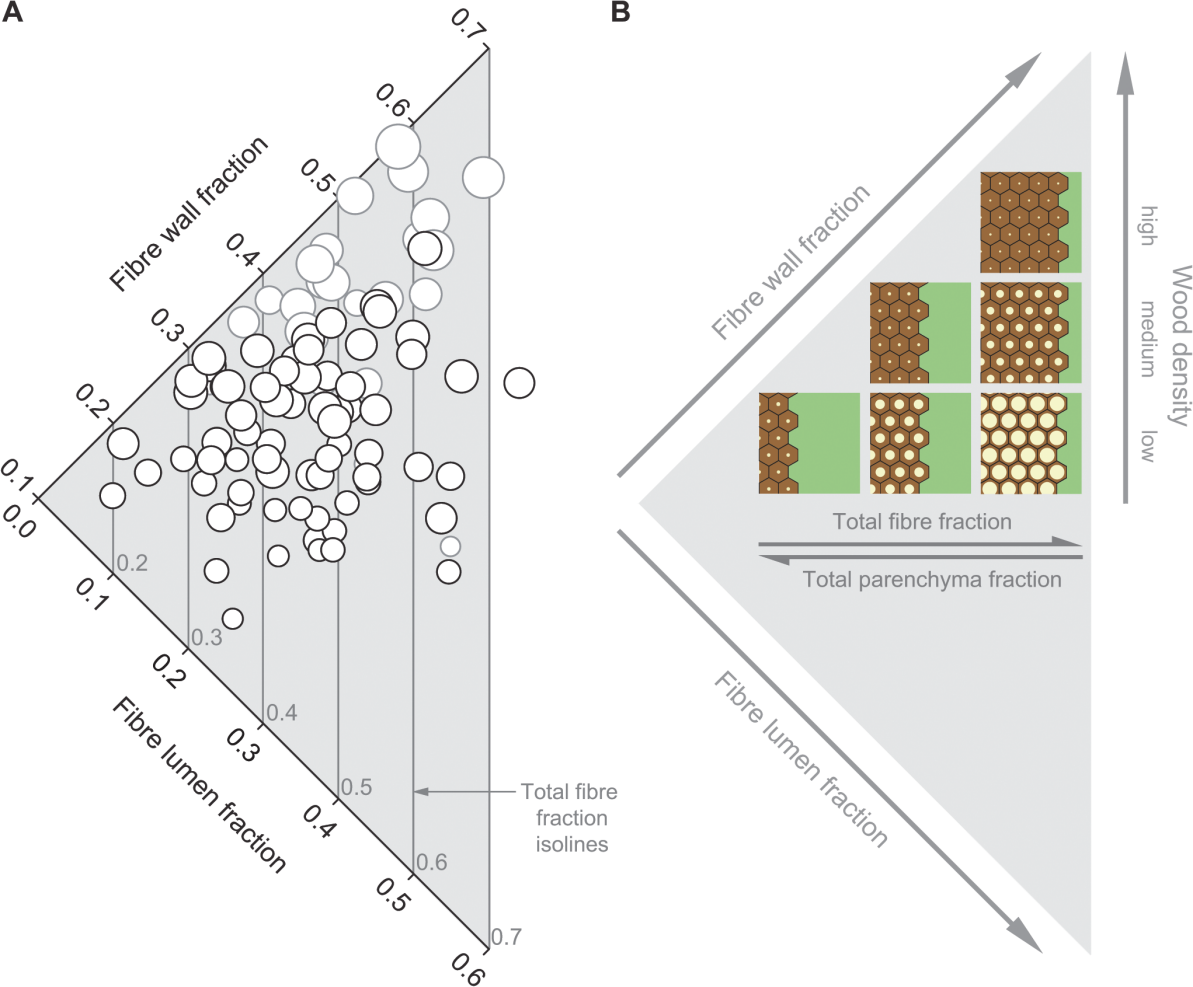
Relationship between fibre wall fraction and fibre lumen fraction across 93 species (A) and a diagram illustrating this relationship (B). (A) Black symbols represent 69 from this study and grey symbols represent 24 species from [12]. Symbol diameter is proportional to species wood density. With the smallest diameter corresponding to the lowest density and the largest diameter to the highest density. Isolines indicate total fibre fraction increasing from left to right by a step of 0.1. Grey numbers above the X axis correspond to total fibre fraction indicated by a given isoline. (B) A diagram illustrating Fig 4A; the diagram is modified from Fig 8 in [12]. The six squares symbolize cross-sections through six different woods. Hexagons are fibres with fibre wall in brown and fibre lumen in bright yellow. Green is parenchyma (axial + ray). Vessel fraction was relatively small, and did not show large variation across species, so for simplicity was omitted in this diagram. Wood density increases towards the top of the diagram. Total fibre fraction (brown wall + yellow lumen) and total parenchyma fraction (green, includes axial + ray) covary negatively with each other and approximately orthogonally to wood density.

Fibre wall fraction and fibre lumen fraction varied independently from each other (species represented by black circles in Fig 4A). However, considered together with species from a previous study, which included higher densities than studied here ([12]; grey circles), the data points were distributed roughly in a triangle (Fig 4A; version of Fig 4A showing site-coded data points as S2 Fig). The highest-density species (largest bubbles, Fig 4A) had large fibre wall fractions and small fibre lumen fractions (top of the graph). As the density decreased, the variability of fibre wall and lumen fractions increased, and many anatomical combinations were observed. For example, a medium fibre wall contribution around 0.30 could be combined either with a large amount of fibre lumen, around 0.30, or with hardly any fibre lumen, below 0.05. Species with only small amounts of both fibre wall and fibre lumen (around 0.20 total) also existed.

Total fibre fraction (isolines in Fig 4A) is the sum of fibre wall and fibre lumen fractions. Total fibre fraction increases from left of the graph (isoline with fraction of 0.2) outwards to isoline with fraction of 0.7. The lowest-density species (smallest bubbles in Fig 4A) varied widely in fibre fraction and the width of this variation decreased towards high-density species (largest bubbles, top of Fig 4A). Also, at any given fibre fraction (along a given isoline) lower density species (smaller bubbles) tended to have higher fibre lumen fraction relative to fibre wall.

As stated above, total fibre fraction was strongly negatively correlated with total parenchyma fraction (Fig 3). Correspondingly, species in left of Fig 4A (low fibre fraction) tended to have high parenchyma fraction. Species positioned along the outward isoline tended to have low parenchyma fraction. Relationships are schematically summarized in Fig 4B, and illustrated with three cross-sections through low-density woods with various anatomies in S3 Fig.

Along with these general trends, inconsistencies were also observed. Species with similar total fibre, fibre wall and fibre lumen fractions sometimes differed in densities (Fig 4A, bubbles that are near to each other but differ in size). For example, two species at the top of Fig 2, *Cardwellia sublimis* and *Argyrodendron peralatum*, had similar structure but moderately different densities (0.47 and 0.58 g cm^-3^, respectively).

Approximate cell wall material density was also calculated and discussed (see S1 Text).

### Anatomical and non-anatomical trait relationships

Our second main objective was to explore potential ecological interpretations of anatomical variation across the studied species. We investigated relationships among parenchyma, vessels, and the three “ecological” traits, leaf area to sapwood area ratio (LA/SA), height (measured height and maximum height sourced from the literature), and modulus of elasticity (MOE; Table 3). The three sampled sites represented tropical rainforest, tropical woodland, and temperate forest (Table 1 and S1 Fig), making some comparisons possible between climates and vegetation types.

**Fig 5:**
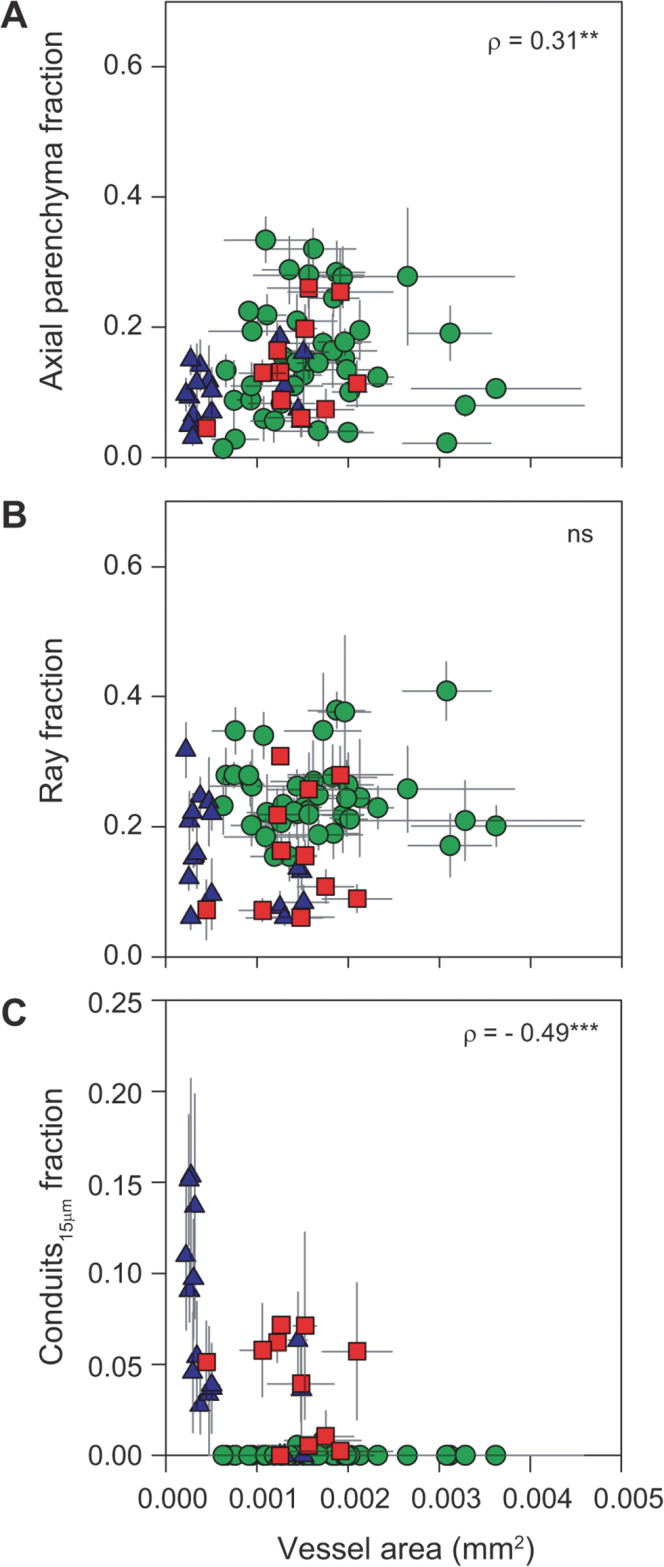
Relationships between axial parenchyma fraction, ray fraction, conduits_15μm_ fraction and vessel area (i.e. cross-sectional average area). Green circles—tropical rainforest (warm and wet site); red squares—tropical woodland (warm and dry site); blue triangles—temperate forest (cool and wet site). * *P* < 0.05, ** *P* < 0.01, ns—not significant.

**Fig 6:**
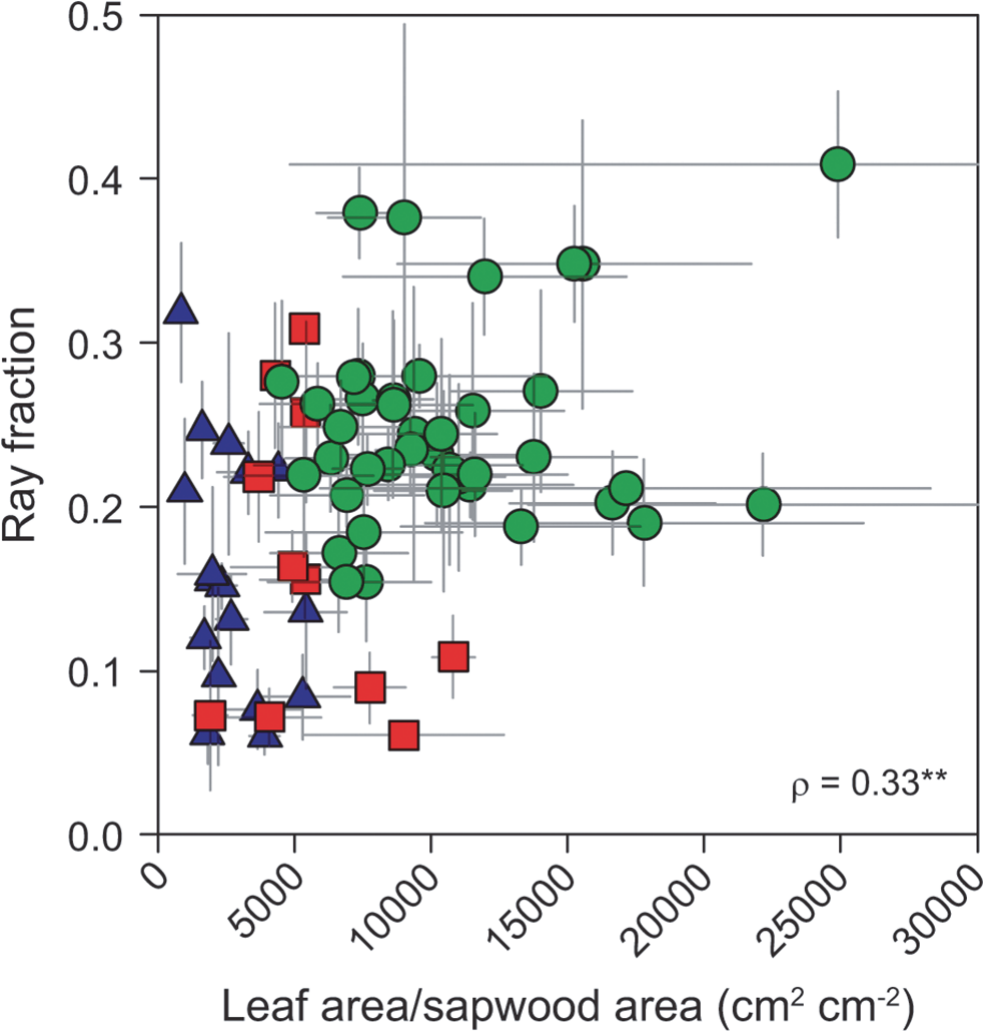
Relationship between ray fraction and leaf area to sapwood area ratio. Green circles—tropical rainforest (warm and wet site); red squares—tropical woodland (warm and dry site); blue triangles—temperate forest (cool and wet site). ** *P* < 0.01.

**Fig 7:**
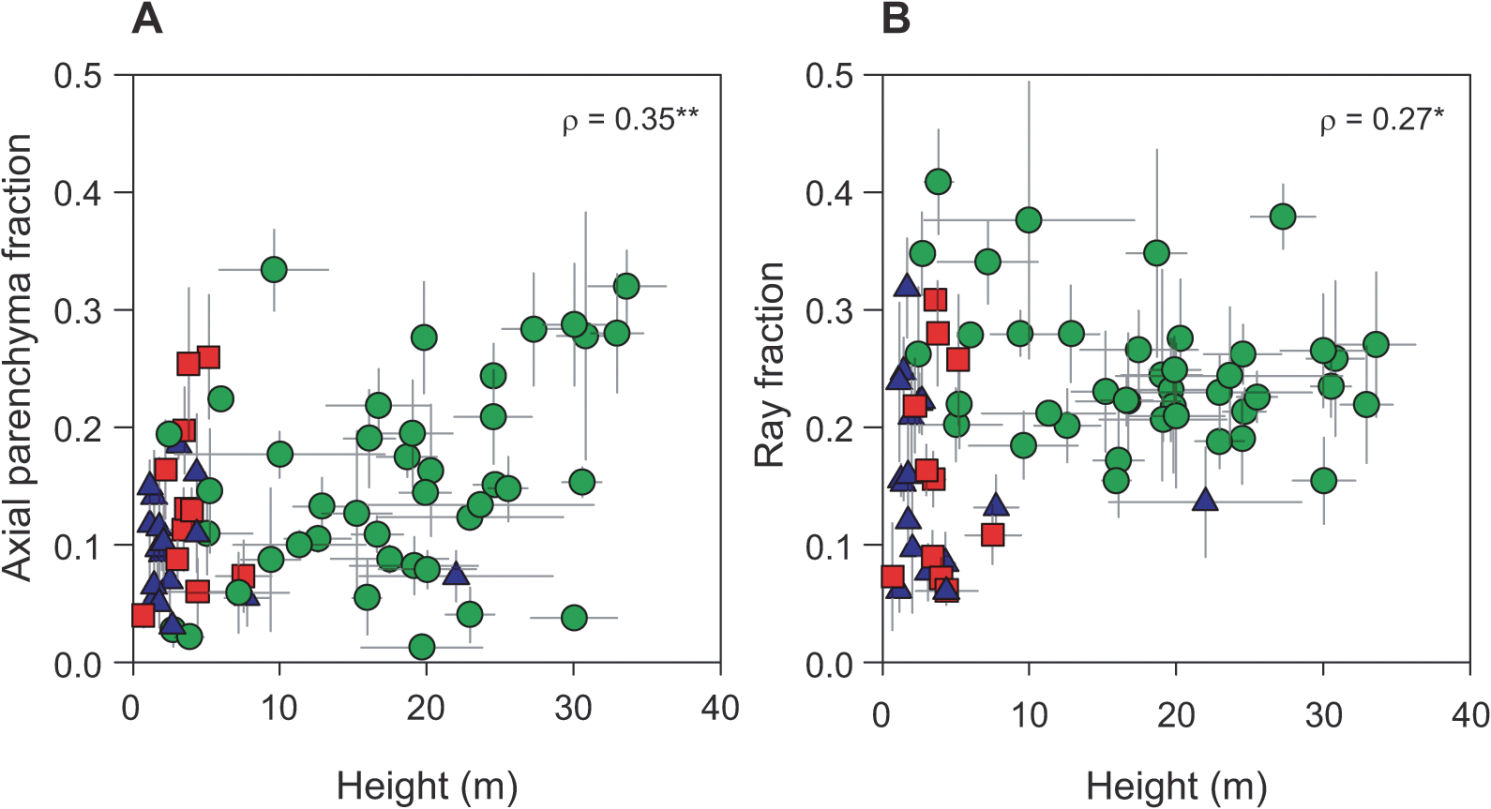
Relationship between axial and ray parenchyma fractions and height. Green circles—tropical rainforest (warm and wet site); red squares—tropical woodland (warm and dry site); blue triangles—temperate forest (cool and wet site). * *P* < 0.05, ** *P* < 0.01.

**Fig 8:**
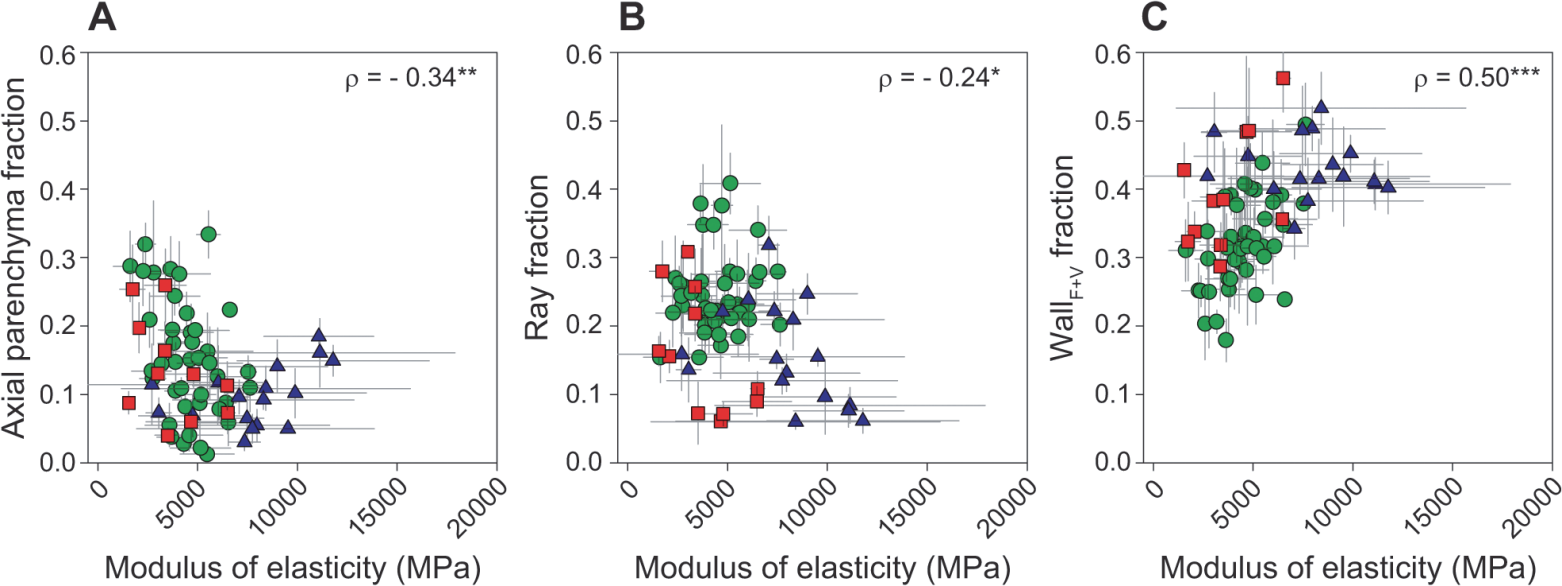
Relationships between axial and ray parenchyma fractions, wall_F+V_ fraction and modulus of elasticity. Each symbol is an individual species. Symbol type represents site of collection: green circles—tropical rainforest (warm and wet site), red squares—tropical woodland (warm and dry site) and blue triangles—temperate forest (cool and wet site). * *P* < 0.05, ** *P* < 0.01, *** *P* < 0.001, **** *P* < 0.0001.

**Fig 9:**
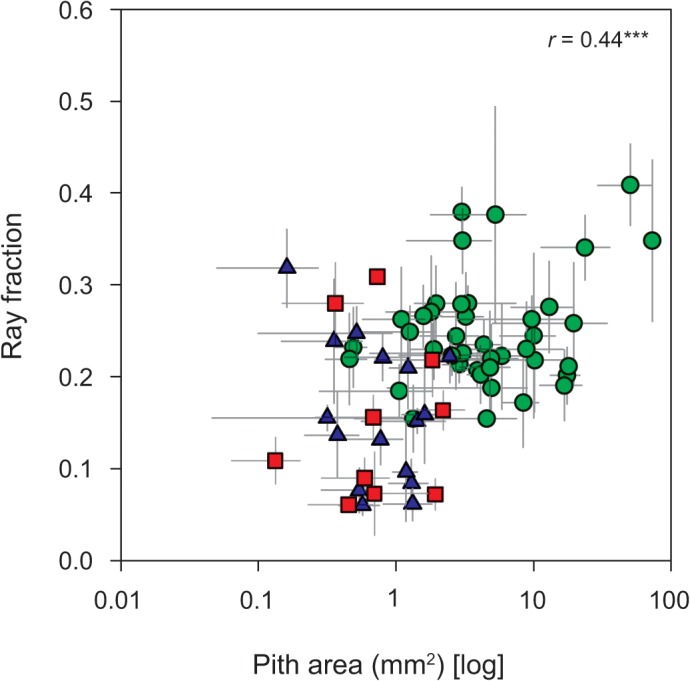
Relationship between ray parenchyma fraction and pith area (log-transformed). Green circles—tropical rainforest (warm and wet site); red squares—tropical woodland (warm and dry site); blue triangles—temperate forest (cool and wet site). *** *P* < 0.001.

Parenchyma and vessel traits were weakly or not at all related with each other, either across all species or within sites. Axial parenchyma fraction tended to increase weakly with each of vessel area, vessel area to number ratio, and hydraulically weighted diameter (*ρ* ranged from 0.29 to 0.36, *P* < 0.05, Table 3 and Fig 5A) and, correspondingly, to decrease with vessel number per area (*ρ* = -0.35, *P* < 0.01). Ray fraction was not associated with vessel properties (Fig 5B). Vessel traits were associated with conduits_15μm_ fraction forming a triangular relationship, which was mostly driven by climate differences (Fig 5C). Species from the tropical rainforest (green circles in Figs 5–9) tended to have large vessels and lack conduits_15μm_ (conduits_15μm_ were observed in only two of 41 species sampled in this site). Species from the tropical woodland (red squares) also tended to have relatively large vessels but conduits_15μm_ were present in 10 of the 11 sampled species, unlike the tropical rainforest. In the temperate forest (blue triangles) vessels were typically small, but accompanied by considerable conduits_15μm_ fractions (present in 14 out of 17 species, the exceptions being three *Acacia* species).

LA/SA was weakly positively correlated with ray fraction (*ρ* = 0.33, *P* < 0.01) but was not related to axial parenchyma fraction. Consequently, the relationship between LA/SA and total parenchyma fraction was similarly weakly positive (Table 3). These weak patterns were driven by site differences: species from the tropical rainforest tended to have higher both LA/SA and ray fractions than in the two other sampled locations (Fig 6). No correlations were found within the tropical woodland nor the temperate forest.

Taller species had a weak tendency to have higher parenchyma fractions (height and maximum height *vs*. axial and ray parenchyma fractions: *ρ* from 0.25 to 0.41, all *P* < 0.05, except there was no correlation between ray fraction and maximum height, Table 3 and Fig 7). These trends are possibly underpinned by the site differences; the tropical rainforest site had more taller and parenchyma rich species than the two other sites. Additionally, within the tropical rainforest, where there was the strongest light gradient, axial parenchyma tended weakly to increase with height and maximum height (*r* = 0.36 and *r* = 0.34, respectively, both *P* < 0.05), while ray and total parenchyma fractions varied independently of height traits. There were no significant relationships within the tropical woodland and the temperate forest.

Species with more elastic twigs (low modulus of elasticity or MOE) tended to have parenchyma-rich woods (total parenchyma, *ρ* = -0.41, *P* < 0.001, axial parenchyma, *ρ* = -0.34, *P* < 0.01, and rays, *ρ* = -0.24, *P* < 0.05, Fig 8A and 8B). However, the strongest anatomical correlate with MOE variation was the amount of fibre and vessel walls (wall_F+V_ fraction, *ρ* = 0.50, *P* < 0.001, Fig 8C), more elastic woods having lower wall fractions. MOE varied weakly with wood density (*ρ* = 0.27, *P* < 0.05), remembering however that only a limited range of wood density was sampled in this study. Within sites, the effects of parenchyma tissues were somewhat different. Axial parenchyma fraction correlated negatively with MOE in the rainforest (*ρ* = -0.35, *P* < 0.05), but was not associated within the two other sites. Ray fraction was correlated negatively in tropical woodland, (*ρ* = -0.63, *P* < 0.05) and temperate forest (*ρ* = -0.59, *P* < 0.05), but not correlated in the rainforest. Interpretation of these results requires extra caution because MOE variation within species was often larger than across species, especially so for the temperate forest site (see error bars in Fig 8). Perhaps the bark, which was left on twigs during MOE measurements, might also have influenced MOE results.

We calculated also a metric indicating leaf mass loading on a twig (leaf mass per area multiplied by leaf area to sapwood area ratio, LMA*LA/SA; LMA was calculated as 1/SLA). This metric was weakly negatively correlated with MOE. Contrary to expectations, twigs with stiffer wood carried lower leaf mass loading (*ρ* = -0.43, *P* < 0.001). However, the relationship was triangular and species with stiff twigs and high leaf mass loading were absent (S4 Fig).

Pith area correlated positively with ray fraction (log-transformed pith area, *r* = - 0.44, *P* < 0.001, Fig 9) across the three sites but not within individual sites. Species from the rainforest tended to have higher pith and ray fractions than in the two other sites. Given the direct physical contact between these two wood components, it is possible they might be functionally interlinked, and this relationship is likely to be climate-dependent.

Site average comparisons are presented in S2 Text. These should be interpreted with great caution because species selection was non-random and there was no site replication within a given climate.

## Discussion

This study aimed to describe anatomical variation approximately orthogonal to wood density, and to explore possible ecological implications for this variation. To our knowledge, this work is the first detailed account of this kind of anatomical variation across a broad sample of angiosperm species.

### The scope of anatomical variation

[12] showed that variation in twig wood anatomy was widest among species with medium wood densities (roughly 0.50–0.75 g cm^-3^). Variation was narrow in species with lower densities (Fig 8 in [12]), but only three species <0.5 g cm^-3^ were sampled in that study. Here we examined 23 species with densities <0.5 g cm^-3^. We found that as foreshadowed in [12], anatomical structure in those 23 species indeed spanned a wide continuum from abundant parenchyma and few fibres to abundant fibres and scarce parenchyma (low-density anatomies in Fig 4 and S3 Fig).

Across all 69 species sampled here there was a wide variation in total parenchyma and total fibre fractions (Figs 2 and 4), and a strong trade-off was present between the two traits (Fig 3). In a series of recent studies this fibre-parenchyma spectrum has been observed across over a thousand angiosperm species from a variety of vegetation types running from tropical to temperate regions [9–13]. Here we found that the span of the fibre-parenchyma spectrum tended to be wider across lower-density species (widening of the triangle in Fig 4B towards lower density). Additionally, for a given total fibre fraction, fibres could be composed of various proportions of wall relative to lumen (Fig 4). This further broadened the possible anatomical options, which are illustrated in Fig 4B (modified from [12]). Examples of wood cross-sections of low-density species are shown in photographs in S3 Fig. Species with densities lower than 0.38 g cm^-3^ (not sampled in this study) could plausibly have even lower fibre wall fraction or higher fibre lumen fraction than found here (the minimum fibre wall fraction reported here was 0.15 and maximum fibre lumen fraction was 0.32). For example, in six baobab species with wood densities 0.09–0.17 g cm^-3^ the solid fraction of wood was only 0.05–0.12 (‘solid fraction’ presumably included all cell walls; [45]). We propose that the fibre-parenchyma spectrum contributes an axis of variation that is largely orthogonal to wood density and that spans wider amplitude towards lower wood density. This implies that there may be substantial ecological strategy variation independent from wood density, especially among lower density species (discussed in more detail below).

In our study, lower-density species (roughly below 0.5 g cm^-3^) were abundant in the rainforest, but not in the two other sites with lower rainfall or temperature. Medium-density species were found in all three sites. This implies that strategies involving large parenchyma or fibre lumen fractions may be not viable or not competitive in certain environments, while anatomical strategies associated with medium-density wood may be ubiquitous.

We also recorded instances where species with similar anatomies varied in their densities, for example *Cardwellia sublimis* and *Argyrodendron peralatum* (Fig 2). This could possibly be explained by differences in starch content. Starch content was not formally quantified, but we rarely observed starch granules in *C*. *sublimis* versus frequently in *A*. *peralatum*. Starch has a density *c*. 1.5 g cm^-3^ [46,47], so the amount of starch could potentially affect overall wood density. Alternatively or additionally to the starch effect, dissimilar densities might have resulted from different fractions of parenchyma wall and/or conduits_15μm_ wall. We did not quantify these fractions, but, for example, across 50 woody angiosperm species the proportion of wall within rays has been found to vary substantially (20 to 70%; [48]).

### Trait comparisons

We hypothesized that species with vessels more prone to embolism might have more parenchyma tissue to participate in embolism repair, especially in sites with longer or more severe stress periods (dry or low temperature seasons). It has been suggested that either axial or ray parenchyma (or both) may facilitate embolism repair and vessel refilling [22–25]. However, few species have been studied; it is not clear how this possibility might relate to the wide variation in parenchyma fraction; and moreover with new technological advances, the prevalence of embolism refilling has recently been questioned [26–30]. In any event, across the species and sites studied here parenchyma fraction was uncorrelated with severity of the dry or cold seasons. Parenchyma characteristics rather than parenchyma quantity might be more important for embolism refilling, if indeed refilling is common at all. The relationships between vessel properties (vessel area, hydraulically weighted diameter, vessel area to number ratio) and parenchyma amounts (fractions of total parenchyma, axial parenchyma, and rays) were weak, either across sites or within individual sites (Fig 5A and 5B). Vessel area and parenchyma properties were similarly unrelated in main stems across a set of species with wider density range ([8–10,13]; 61, 42, 111, and 113 species, respectively, from tropical and subtropical zones). In addition, vessel area has been shown to vary independently from wood density or with weak negative correlation [6,8–10,12,13,49–51]. Thus, a vessel area axis of variation seems to be substantially orthogonal to the wood density and the fibre-parenchyma axes of variation.

Very small vessels or tracheids (here ‘conduits_15μm_‘ meaning conduits with maximum lumen diameter below 15 μm) could also play an auxiliary role during drought or freeze-thaw events, for example, in the tropical woodland or the temperate forest studied here. They were present in most species from those two sites but in hardly any species from the tropical rainforest.

At least in cacti, Crassulaceae and baobabs, parenchyma may serve as a storage compartment for water [17,45]; however it is unclear whether this might also be true for species with less unusual wood anatomies (and propensity for stem water storage). It has been also shown that lower-density species tend to have higher capacitance [52–56] but it is not known whether that capacitance should be attributed to parenchyma fraction or rather to fibre lumen fraction. It is not yet well understood how capacitance from parenchyma would work; according to [17] water release from parenchyma may require whole stem volume adjustments and be challenging. Possibly, abundant parenchyma, well connected in three-dimensional space, could achieve the required volume adjustments. Joint capacitance and parenchyma studies could be helpful in understanding parenchyma’s role in water storage.

Before this research we hypothesised that shade-tolerant species in the rainforest, a site with a strong light gradient, would have higher parenchyma fraction. This hypothesis arose from the idea that more parenchyma represented higher capacity to store metabolites, and also from the finding that higher carbohydrate storage tended to increase seedling survival during light stress in seven rainforest species [57]. We found the opposite trend from this prediction, although weakly: axial parenchyma fraction tended to increase with maximum height within the rainforest (*ρ* = 0.34, *P* < 0.05), while ray fraction varied independently. What might be causing this contradiction? Firstly, perhaps tall species need parenchyma for predominantly different purposes compared to short species. For example, taller species being more exposed to winds might build twigs that are more elastic. Here, tall species in the rainforest site tended to be more elastic (lower MOE; *ρ* = -0.62, *P* < 0.001). And this lower MOE could be partly due to higher axial parenchyma fraction (MOE *vs*. axial parenchyma fraction, *ρ* = -0.35, *P* < 0.05). Secondly, perhaps the premise that high parenchyma fraction corresponds to high metabolite storage is not always valid. Parenchyma might be a reservoir of capacitance water, as mentioned above, or play a dual role as a reservoir of both water and carbohydrates.

Although the role of parenchyma in carbohydrate storage is well proven [16,31], we are not aware of quantitative data demonstrating association between the fraction of parenchyma and the quantity of stored carbohydrates across a broad range of woody angiosperms. Since stored carbohydrates are being withheld from commitment to growth, storage is not self-evidently an advantage; rather its benefits and costs need to be considered in particular climate contexts. High parenchyma fractions presumably incur higher maintenance costs, and those costs might be less affordable in water or temperature stressed environments (here the tropical woodland and the temperate forest). Studies relating maintenance costs of wood (e.g. measured as respiration) with parenchyma fraction and carbohydrate storage could be of great help in understanding wood anatomy and its ecological implications.

Finally, we asked how parenchyma traits might affect MOE across all studied species. It has been shown across large datasets that wood density is positively correlated with MOE [4]. However, there is also substantial variation in mechanical properties that is independent of wood density [38,58], and likely is influenced by anatomical structure [58]. We found that across all species, wall_F+V_ fraction (fibre wall + vessel wall) had the strongest effect on MOE variation (positive relationship), while parenchyma amounts had secondary effects (weak, negative relationships). The weak correlation between parenchyma and MOE may have several causes. Firstly, the influence of parenchyma on mechanical properties might depend on the proportion of wall within parenchyma fraction. Ray parenchyma, at least, has a variable proportion of wall (20–70% across 50 Japanese angiosperm trees [48]). Secondly, other parenchyma properties such as the geometry of the tissue and the cells may play a more important role in wood elasticity than tissue fraction. For example, axial parenchyma can be distributed in a diffuse or aggregate pattern, and rays can have variable width, height, and number per mm. Ray width was positively related to radial MOE and ray fraction was positively related to tangential MOE in isolated wood blocks of eight angiosperm trees [59]. Tensile strength of individual large beech rays isolated from wood has been shown to reach around 80 MPa [60], so a few tens of rays could make a considerable contribution to MOE (lowest MOE measured here approximately 1500 MPa). The variation in ray geometry and ray wall fraction can also possibly explain why ray fraction was negatively correlated with MOE in our study, but positively correlated in a previous study of five *Acer* species with similar absolute wood density range (0.47 to 0.72 g cm^-3^; [37]).

It is noteworthy that the fraction of parenchyma might not be a single, decisive trait determining parenchyma functions. Other parenchyma characteristics—for instance, distribution patterns of parenchyma cells within xylem and their location relative to other cells, parenchyma cell shape and wall thickness, amount of shared border with other cell types, parenchyma-vessel pit size and density, or fine scale details such as the amount of plasmodesmata per pit area—may play an important role.

### Conclusions

In this study, we draw attention to substantial variation in anatomical structure that is largely independent of wood density and has been little discussed in the trait ecology literature. We suggest that there are at least three dimensions of variation that are substantially independent of each other, meaning that all sorts of combinations among them can be found: 1) wood density variation, mainly driven by fibre wall and lumen fractions [5–8,12,13], 2) a fibre-parenchyma axis of variation, with the breadth of variation increasing towards lower density species, and 3) a vessel area dimension. Among these, the vessel area dimension is relatively well understood and indicates water conductive safety and efficiency strategies [61].

In this work, we sought potential ecological correlates of the fibre-parenchyma dimension of variation. This dimension proved to be very weakly or not at all correlated with the other traits measured, nor with the climate at different sites. Consequently, the ecological significance of fibre-parenchyma variation remains unclear. Nevertheless, this study makes a valuable contribution by describing quantitatively this intriguing anatomical variation, by rejecting some of the possible hypotheses about what it might be correlated with, and by indicating paths for future research.

## Supporting Information

S1 Fig Study sites. The sites represent three different climates and vegetation types: tropical rainforest (Cape Tribulation, Daintree National Park, QLD), tropical woodland (Blencoe Falls, Girringun National Park, QLD), and temperate forest (Thredbo, Kosciuszko National Park, NSW).
(TIF)

S2 Fig Relationship between fibre wall fraction and fibre lumen fraction across 93 species showing site-coded data points. Each symbol represents one species. Different symbols correspond to sites: green circles—tropical rainforest, red squares—tropical woodland, blue triangles—temperate forest. White diamonds are species from Ziemińska et al. (2013).
(EPS)

S3 Fig Cross-sections through twigs of three low-density species. These approximately correspond to the three low-density anatomies in Fig 4B (diagrams repeated here below images). *Gomphandra australiana* to the left (wood density 0.47 g cm^-3^), *Dysoxylum arborescens* in the middle (wood density 0.53 g cm^-3^) and *Elaeocarpus grandis* to the right (wood density 0.44 g cm^-3^). All three species were sampled in the tropical rainforest (Cape Tribulation). V—vessels, FL—fibre lumen, FW—fibre wall, A—axial parenchyma, R—ray parenchyma. Note starch granules are faintly noticeable in parenchyma of *D*. *arborescens*. Axial parenchyma in *E*. *grandis* is hardly discernible at this resolution (but higher resolution photos were used for image analysis and axial parenchyma was possible to identify). Tissue fractions of each corresponding species are listed below the images. Scale bar corresponds to 100 μm.
(TIF)

S4 Fig Relationship between LMA*LA/SA and modulus of elasticity. LMA—leaf mass per area, LA/SA—leaf area to sapwood area ratio. Each symbol represents one species. Different symbols correspond to sites: green circles—tropical rainforest, red squares—tropical woodland, blue triangles—temperate forest.
(EPS)

S1 Table Species and families. (DOCX)

S2 Table Dataset. (XLSX)

S1 Text Cell wall material density note. (DOCX)

S2 Text Site comparisons note. (DOCX)

## References

[1] Grubb PJ. A reassessment of the strategies of plants which cope with shortages of resources. Perspect Plant Ecol Evol Syst. 1998;1: 3–31. 10.1078/1433-8319-00049

[2] Westoby M, Falster DS, Moles AT, Vesk PA, Wright IJ. Plant ecological strategies: some leading dimensions of variation between species. Annu Rev Ecol Syst. 2002;33: 125–159.

[3] Grime JP. Plant Strategies, Vegetation Processes, and Ecosystem Properties. John Wiley & Sons; 2006.

[4] Chave J, Coomes D, Jansen S, Lewis SL, Swenson NG, Zanne AE. Towards a worldwide wood economics spectrum. Ecol Lett. 2009;12: 351–366. 10.1111/j.1461-0248.2009.01285.x 19243406

[5] Fujiwara S, Sameshima K, Kuroda K, Takamura N. Anatomy and properties of Japanese hardwoods. I. Variation of fibre dimensions and tissue proportions and their relation to basic density. IAWA Bull Ns. 1991;12: 419–24.

[6] Jacobsen AL, Agenbag L, Esler KJ, Pratt RB, Ewers FW, Davis SD. Xylem density, biomechanics and anatomical traits correlate with water stress in 17 evergreen shrub species of the Mediterranean‐type climate region of South Africa. J Ecol. 2007;95: 171–183. 10.1111/j.1365-2745.2006.01186.x

[7] Rana R, Langenfeld-Heyser R, Finkeldey R, Polle A. Functional anatomy of five endangered tropical timber wood species of the family Dipterocarpaceae. Trees—Struct Funct. 2009;23: 521–529. 10.1007/s00468-008-0298-4

[8] Martínez-Cabrera HI, Jones CS, Espino S, Schenk HJ. Wood anatomy and wood density in shrubs: responses to varying aridity along transcontinental transects. Am J Bot. 2009;96: 1388–1398. 10.3732/ajb.0800237 21628286

[9] Poorter L, McDonald I, Alarcón A, Fichtler E, Licona J, Peña‐Claros M, et al The importance of wood traits and hydraulic conductance for the performance and life history strategies of 42 rainforest tree species. New Phytol. 2010;185: 481–492. 10.1111/j.1469-8137.2009.03092.x 19925555

[10] Fichtler E, Worbes M. Wood anatomical variables in tropical trees and their relation to site conditions and individual tree morphology. IAWA J. 2012;33: 119–140.

[11] Zheng J, Martínez-Cabrera HI. Wood anatomical correlates with theoretical conductivity and wood density across China: evolutionary evidence of the functional differentiation of axial and radial parenchyma. Ann Bot. 2013;112: 927–935. 10.1093/aob/mct153 23904446 PMC3747806

[12] Ziemińska K, Butler DW, Gleason SM, Wright IJ, Westoby M. Fibre wall and lumen fractions drive wood density variation across 24 Australian angiosperms. AoB PLANTS. 2013;5 10.1093/aobpla/plt046

[13] Fortunel C, Ruelle J, Beauchêne J, Fine PVA, Baraloto C. Wood specific gravity and anatomy of branches and roots in 113 Amazonian rainforest tree species across environmental gradients. New Phytol. 2014;202: 79–94. 10.1111/nph.12632 24329812

[14] Zanne AE, Westoby M, Falster DS, Ackerly DD, Loarie SR, Arnold SEJ, et al Angiosperm wood structure: global patterns in vessel anatomy and their relation to wood density and potential conductivity. Am J Bot. 2010;97: 207–215. 10.3732/ajb.0900178 21622380

[15] Global Wood Density Database [Internet]. 4 Feb 2009. Available: http://hdl.handle.net/10255/dryad.235

[16] Evert RF. Esau’s plant anatomy: meristems, cells, and tissues of the plant body: their structure, function, and development Hoboken, New Jersey: John Wiley & Sons; 2006.

[17] Holbrook NM. Stem water storage. Plant stems: physiology and functional morphology Academic Press; 1995 pp. 151–174.

[18] Zwieniecki MA, Melcher PJ, Ahrens ET. Analysis of spatial and temporal dynamics of xylem refilling in Acer rubrum L. using magnetic resonance imaging. Front Plant Sci. 2013;4 10.3389/fpls.2013.00265 23885258 PMC3717611

[19] Brodersen CR, McElrone AJ, Choat B, Matthews MA, Shackel KA. The Dynamics of Embolism Repair in Xylem: In Vivo Visualizations Using High-Resolution Computed Tomography. Plant Physiol. 2010;154: 1088–1095. 10.1104/pp.110.162396 20841451 PMC2971590

[20] Trifilò P, Raimondo F, Lo Gullo MA, Barbera PM, Salleo S, Nardini A. Relax and refill: xylem rehydration prior to hydraulic measurements favours embolism repair in stems and generates artificially low PLC values. Plant Cell Environ. 2014;37: 2491–2499. 10.1111/pce.12313 24588546

[21] Trifilò P, Barbera PM, Raimondo F, Nardini A, Gullo MAL. Coping with drought-induced xylem cavitation: coordination of embolism repair and ionic effects in three Mediterranean evergreens. Tree Physiol. 2014;34: 109–122. 10.1093/treephys/tpt119 24488800

[22] Nardini A, Lo Gullo MA, Salleo S. Refilling embolized xylem conduits: Is it a matter of phloem unloading? Plant Sci. 2011;180: 604–611. 10.1016/j.plantsci.2010.12.011 21421408

[23] Améglio T, Decourteix M, Alves G, Valentin V, Sakr S, Julien J-L, et al Temperature effects on xylem sap osmolarity in walnut trees: evidence for a vitalistic model of winter embolism repair. Tree Physiol. 2004;24: 785–793. 10.1093/treephys/24.7.785 15123450

[24] Secchi F, Zwieniecki MA. Sensing embolism in xylem vessels: the role of sucrose as a trigger for refilling. Plant Cell Environ. 2011;34: 514–524. 10.1111/j.1365-3040.2010.02259.x 21118423

[25] Salleo S, Lo Gullo MA, Trifilò P, Nardini A. New evidence for a role of vessel-associated cells and phloem in the rapid xylem refilling of cavitated stems of *Laurus nobilis* L. Plant Cell Environ. 2004;27: 1065–1076. 10.1111/j.1365-3040.2004.01211.x

[26] Wheeler JK, Huggett BA, Tofte AN, Rockwell FE, Holbrook NM. Cutting xylem under tension or supersaturated with gas can generate PLC and the appearance of rapid recovery from embolism. Plant Cell Environ. 2013;36: 1938–1949. 10.1111/pce.12139 23701011

[27] Sperry J. Cutting-edge research or cutting-edge artefact? An overdue control experiment complicates the xylem refilling story. Plant Cell Environ. 2013;36: 1916–1918. 10.1111/pce.12148 23763611

[28] Delzon S, Cochard H. Recent advances in tree hydraulics highlight the ecological significance of the hydraulic safety margin. New Phytol. 2014;203: 355–358. 10.1111/nph.12798 24661229

[29] Torres-Ruiz JM, Jansen S, Choat B, McElrone AJ, Cochard H, Brodribb TJ, et al Direct X-Ray Microtomography Observation Confirms the Induction of Embolism upon Xylem Cutting under Tension. Plant Physiol. 2015;167: 40–43. 10.1104/pp.114.249706 25378693 PMC4281005

[30] Cochard H, Delzon S, Badel E. X-ray microtomography (micro-CT): a reference technology for high-resolution quantification of xylem embolism in trees. Plant Cell Environ. 2015;38: 201–206. 10.1111/pce.12391 24942003

[31] Spicer R. Symplasmic networks in secondary vascular tissues: parenchyma distribution and activity supporting long-distance transport. J Exp Bot. 2014; ert459. 10.1093/jxb/ert459 24453225

[32] Romero C, Bolker BM. Effects of stem anatomical and structural traits on responses to stem damage: an experimental study in the Bolivian Amazon. Can J For Res. 2008;38: 611–618.

[33] Shigo AL. Compartmentalization: A Conceptual Framework for Understanding How Trees Grow and Defend Themselves. Annu Rev Phytopathol. 1984;22: 189–214. 10.1146/annurev.py.22.090184.001201

[34] Chattaway M. The Development of Tyloses and Secretion of Gum in·Heartwood Formation. Aust J Biol Sci. 1949;2: 227–240.

[35] Zimmermann MH. The discovery of tylose formatin by a Viennese lady in 1845. IAWA Bull Ns. 1979;2–3: 51–56.

[36] IAWA Committee. IAWA list of microscopic features for hardwood identification. IAWA Bull Ns. 1989;10: 219–332.

[37] Woodrum CL, Ewers FW, Telewski FW. Hydraulic, biomechanical, and anatomical interactions of xylem from five species of *Acer* (Aceraceae). Am J Bot. 2003;90: 693–699. 10.3732/ajb.90.5.693 21659164

[38] Onoda Y, Richards AE, Westoby M. The relationship between stem biomechanics and wood density is modified by rainfall in 32 Australian woody plant species. New Phytol. 2010;185: 493–501. 10.1111/j.1469-8137.2009.03088.x 19925557

[39] Rosell JA, Gleason S, Méndez-Alonzo R, Chang Y, Westoby M. Bark functional ecology: evidence for tradeoffs, functional coordination, and environment producing bark diversity. New Phytol. 2014;201: 486–497. 10.1111/nph.12541 24117609

[40] Grote K-H, Antonsson EK. Springer Handbook of Mechanical Engineering. Springer; 2009.

[41] Carlquist S. Comparative wood anatomy: systematic, ecological, and evolutionary aspects of dicotyledon wood Springer; 2001.

[42] Gerlach D. Zarys mikrotechniki botanicznej Warszawa: Państwowe Wydawnictwo Rolnicze i Leśne; 1972.

[43] Smith DM. Microscopic methods for determining cross-sectional cell dimensions. US For Serv Res Pap FPL 79 1967;

[44] Sperry JS, Nichols KL, Sullivan JEM, Eastlack SE. Xylem embolism in ring-porous, diffuse-porous, and coniferous trees of Northern Utah and Interior Alaska. Ecology. 1994;75: 1736–1752. 10.2307/1939633

[45] Chapotin SM, Razanameharizaka JH, Holbrook NM. A biomechanical perspective on the role of large stem volume and high water content in baobab trees (*Adansonia* spp.; Bombacaceae). Am J Bot. 2006;93: 1251–1264. 10.3732/ajb.93.9.1251 21642189

[46] Gordon R. A retaliatory role for algal projectiles, with implications for the mechanochemistry of diatom gliding motility. J Theor Biol. 1987;126: 419–436. 10.1016/S0022-5193(87)80149-2

[47] Rodriguez-Perez MA, Simoes RD, Constantino CJL, de Saja JA. Structure and physical properties of EVA/starch precursor materials for foaming applications. J Appl Polym Sci. 2011;121: 2324–2330. 10.1002/app.33946

[48] Fujiwara S. Anatomy and properties of Japanese hardwoods II. Variation of dimensions of ray cells and their relation to basic density. IAWA Bull Ns. 1992;13: 397–402.

[49] Mitchell PJ, Veneklaas EJ, Lambers H, Burgess SSO. Using multiple trait associations to define hydraulic functional types in plant communities of south-western Australia. Oecologia. 2008;158: 385–397. 10.1007/s00442-008-1152-5 18839215

[50] Martínez-Cabrera HI, Schenk HJ, Cevallos-Ferriz SRS, Jones CS. Integration of vessel traits, wood density, and height in angiosperm shrubs and trees. Am J Bot. 2011;98: 915–922. 10.3732/ajb.1000335 21613189

[51] Fan Z-X, Zhang S-B, Hao G-Y, Ferry Slik JW, Cao K-F. Hydraulic conductivity traits predict growth rates and adult stature of 40 Asian tropical tree species better than wood density. J Ecol. 2012;100: 732–741. 10.1111/j.1365-2745.2011.01939.x

[52] Meinzer FC, James SA, Goldstein G, Woodruff D. Whole-tree water transport scales with sapwood capacitance in tropical forest canopy trees. Plant Cell Environ. 2003;26: 1147–1155. 10.1046/j.1365-3040.2003.01039.x

[53] Meinzer FC, Campanello PI, Domec J-C, Gatti MG, Goldstein G, Villalobos-Vega R, et al Constraints on physiological function associated with branch architecture and wood density in tropical forest trees. Tree Physiol. 2008;28: 1609–1617. 10.1093/treephys/28.11.1609 18765366

[54] Scholz FG, Bucci SJ, Goldstein G, Meinzer FC, Franco AC, Miralles-Wilhelm F. Biophysical properties and functional significance of stem water storage tissues in Neotropical savanna trees. Plant Cell Environ. 2007;30: 236–248. 10.1111/j.1365-3040.2006.01623.x 17238914

[55] Pratt RB, Jacobsen AL, Ewers FW, Davis SD. Relationships among xylem transport, biomechanics and storage in stems and roots of nine Rhamnaceae species of the California chaparral. New Phytol. 2007;174: 787–798. 10.1111/j.1469-8137.2007.02061.x 17504462

[56] Richards AE, Wright IJ, Lenz TI, Zanne AE. Sapwood capacitance is greater in evergreen sclerophyll species growing in high compared to low-rainfall environments. Funct Ecol. 2014;28: 734–744. 10.1111/1365-2435.12193

[57] Myers JA, Kitajima K. Carbohydrate storage enhances seedling shade and stress tolerance in a neotropical forest. J Ecol. 2007;95: 383–395. 10.1111/j.1365-2745.2006.01207.x

[58] Hepworth DG, Vincent JFV, Stringer G, Jeronimidis G. Variations in the morphology of wood structure can explain why hardwood species of similar density have very different resistances to impact and compressive loading. Philos Trans R Soc Lond Ser Math Phys Eng Sci. 2002;360: 255–272. 10.1098/rsta.2001.0927 16210180

[59] Beery WH, Ifju G, McLain TE. Quantitative wood anatomy—relating anatomy to transverse tensile strength. Wood Fiber Sci. 1983;15: 395–407.

[60] Burgert I, Eckstein D. The tensile strength of isolated wood rays of beech (*Fagus sylvatica* L.) and its significance for the biomechanics of living trees. Trees. 2001;15: 168–170. 10.1007/s004680000086

[61] Tyree MT, Zimmermann MH. Xylem structure and the ascent of sap Springer; 2002.

