# Correction: Broad Anatomical Variation within a Narrow Wood Density Range—A Study of Twig Wood across 69 Australian Angiosperms

**DOI:** 10.1371/journal.pone.0139496

**Published:** 2015-09-25

**Authors:** Kasia Ziemińska, Mark Westoby, Ian J. Wright

The “Hydraulically weighted diameter (mm)—average” and “Hydraulically weighted diameter (mm) –standard deviation” columns in S2 Table are incorrect. Please see the correct S2 Table below.

## Supporting Information

S2 Table Dataset. (XLSX)
