# Supplementary figures and images for: Broad Anatomical Variation within a Narrow Wood Density Range—A Study of Twig Wood across 69 Australian Angiosperms
Source: PLoS One. 2015 Apr 23;10(4):e0124892. doi: 10.1371/journal.pone.0124892 (PMC4408027; doi:10.1371/journal.pone.0124892)

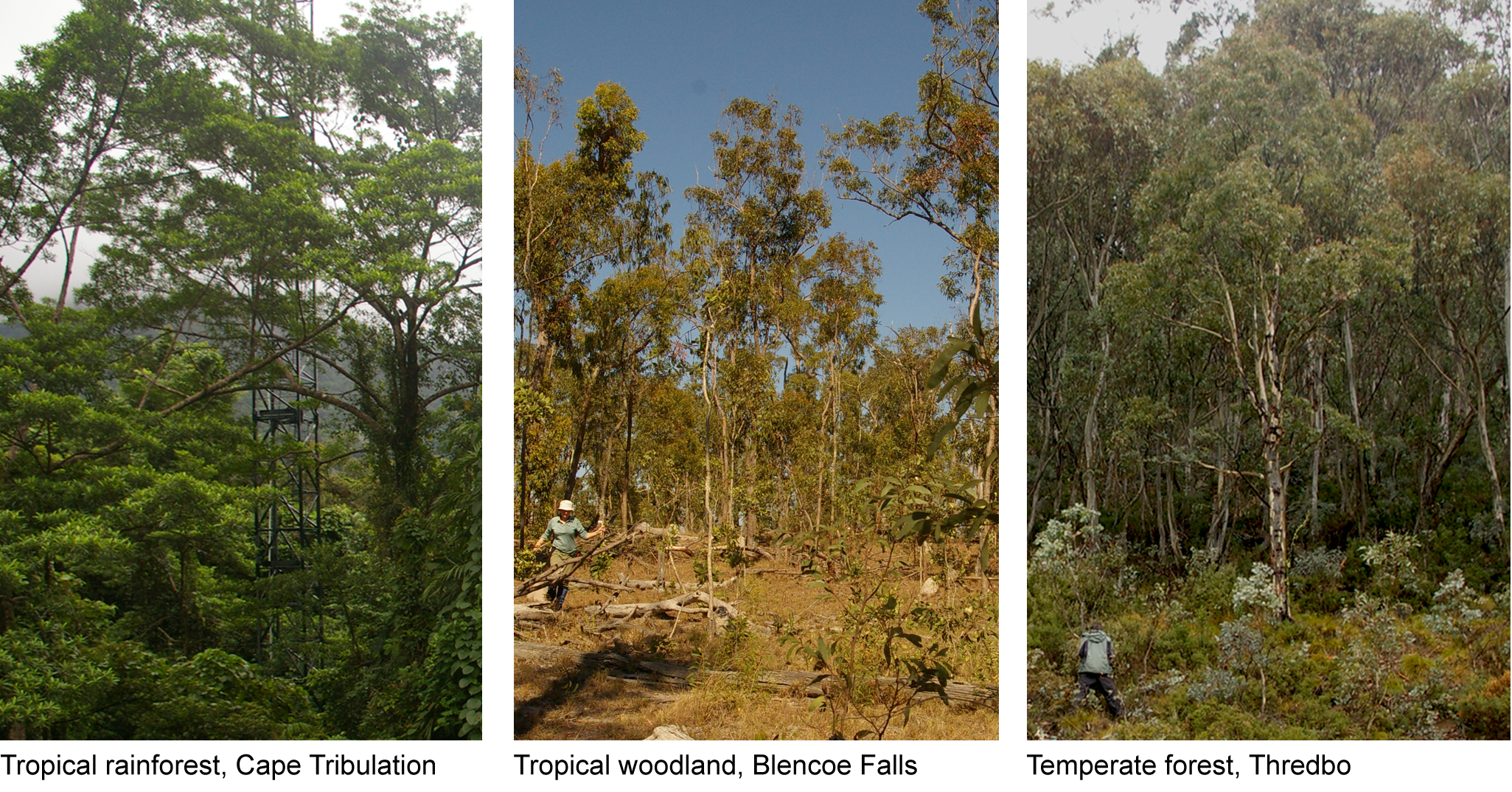

Supplement: S1 Fig — The sites represent three different climates and vegetation types: tropical rainforest (Cape Tribulation, Daintree National Park, QLD), tropical woodland (Blencoe Falls, Girringun National Park, QLD), and temperate forest (Thredbo, Kosciuszko National Park, NSW). (TIF) [file pone.0124892.s001.tif]

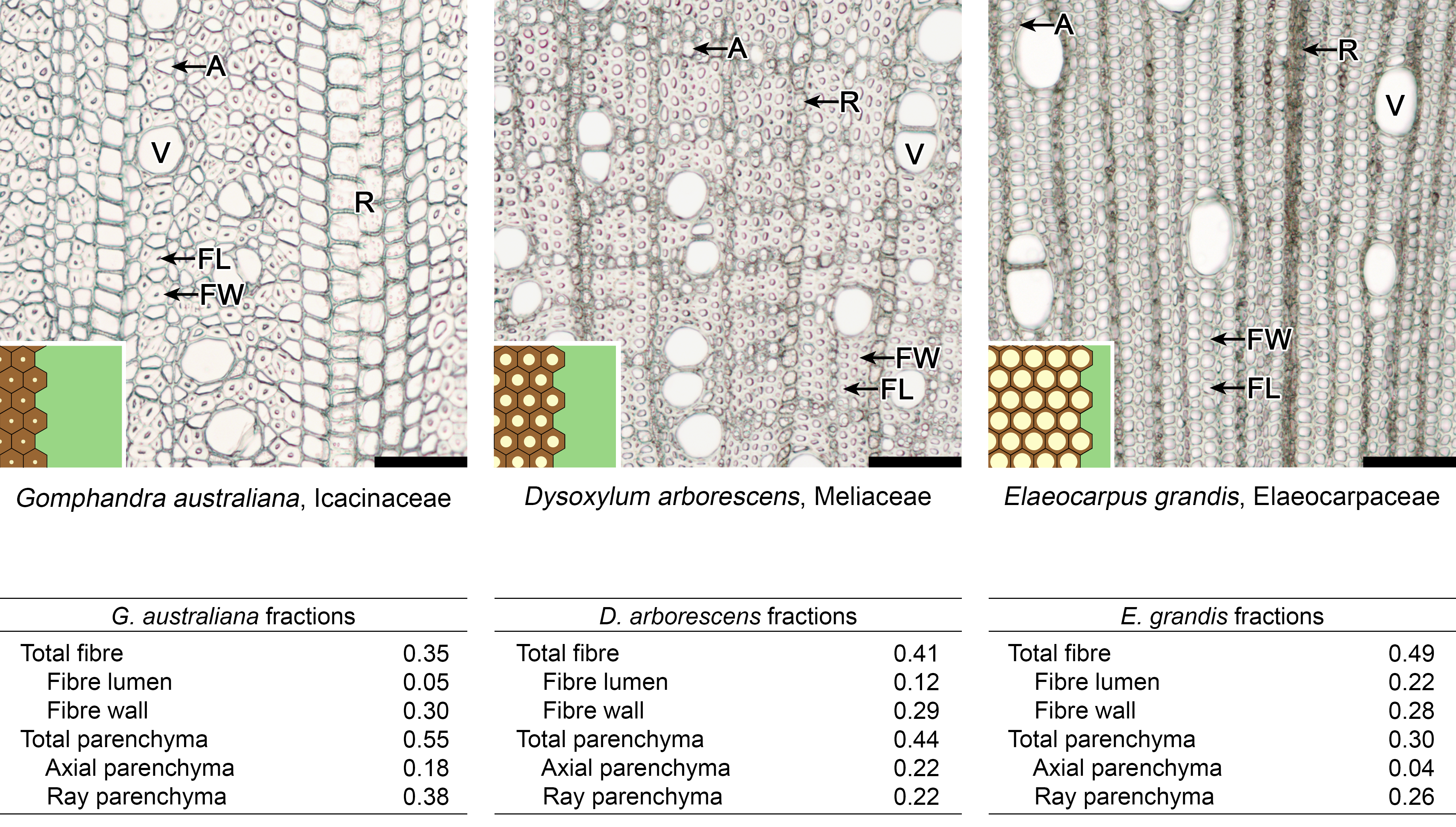

Supplement: S3 Fig — These approximately correspond to the three low-density anatomies in Fig 4B (diagrams repeated here below images). Gomphandra australiana to the left (wood density 0.47 g cm-3), Dysoxylum arborescens in the middle (wood density 0.53 g cm-3) and Elaeocarpus grandis to the right (wood density 0.44 g cm-3). All three species were sampled in the tropical rainforest (Cape Tribulation). V—vessels, FL—fibre lumen, FW—fibre wall, A—axial parenchyma, R—ray parenchyma. Note starch granules are faintly noticeable in parenchyma of D. arborescens. Axial parenchyma in E. grandis is hardly discernible at this resolution (but higher resolution photos were used for image analysis and axial parenchyma was possible to identify). Tissue fractions of each corresponding species are listed below the images. Scale bar corresponds to 100 μm. (TIF) [file pone.0124892.s003.tif]
