# Supplementary material for: Broad Anatomical Variation within a Narrow Wood Density Range—A Study of Twig Wood across 69 Australian Angiosperms
Source: PLoS One. 2015 Apr 23;10(4):e0124892. doi: 10.1371/journal.pone.0124892 (PMC4408027; doi:10.1371/journal.pone.0124892)
Supplement: S1 Table — (DOCX) [file pone.0124892.s005.docx]

Table S1 Species and families.

| Cape Tribulation, tropical rainforest | | Blencoe Falls, tropical woodland | |
| --- | --- | --- | --- |
| Family | Species | Family | Species |
| Annonaceae | Haplostichanthus ramiflorus | Myrtaceae | Corymbia citriodora |
| Apocynaceae | Wrightia laevis |  | Corymbia clarksoniana |
| Cunnoniaceae | Gillbeea whypallana |  | Eucalyptus sp. |
| Elaeocarpaceae | Elaeocarpus grandis |  | Lophostemon suaveolens |
| Euphorbiaceae | Cleistanthus myrianthus |  | Melaleuca nervosa |
|  | Mallotus paniculatus |  | Melaleuca viridiflora |
|  | Rockinghamia angustifolia | Proteaceae | Grevillea glauca |
| Eupomatiaceae | Eupomatia laurina |  | Grevillea parallela |
| Fabaceae | Castanospermum australe |  | Persoonia falcata |
| Hernandiaceae | Hernandia albiflora |  | Xylomelum scottianum |
| Icacinaceae | Gomphandra australiana | Thymelaeaceae | Pimelea linifolia |
| Lauraceae | Cryptocarya grandis | Thredbo, temperate forest | |
|  | Cryptocarya mackinnoniana | Family | Species |
|  | Cryptocarya murrayi | Araliaceae | Polyscias sambucifolia |
|  | Endiandra leptodendron | Asteraceae | Olearia megalophylla |
|  | Endiandra microneura |  | Olearia phlogopappa |
|  | Litsea leefeana |  | Ozothamnus secundiflorus |
| Meliaceae | Dysoxylum alliaceum | Fabaceae | Acacia dealbata |
|  | Dysoxylum arborescens |  | Acacia melanoxylon |
|  | Dysoxylum papuanum |  | Acacia obliquinervia |
|  | Dysoxylum parasiticum | Myrtaceae | Eucalyptus pauciflora |
|  | Dysoxylum pettigrewianum |  | Eucalyptus sp. |
| Monimiaceae | Doryphora aromatica | Proteaceae | Hakea lissosperma |
| Moraceae | Ficus variegata |  | Hakea microcarpa |
| Myristicaceae | Myristica globosa |  | Lomatia myricoides |
| Myrtaceae | Syzygium graveolens |  | Persoonia subvelutina |
|  | Syzygium monospermum | Rubiaceae | Coprosma hirtella |
|  | Syzygium sayeri | Rutaceae | Leionema phylicifolium |
| Proteaceae | Austromuellera trinervia | Santalaceae | Exocarpos strictus |
|  | Cardwellia sublimis | Thymelaeaceae | Pimelea linifolia |
|  | Musgravea heterophylla |  |  |
| Rubiaceae | Antirhea tenuiflora |  |  |
| Rutaceae | Brombya platynema |  |  |
|  | Melicope xanthoxyloides |  |  |
| Salicaceae | Casearia dallachii |  |  |
| Sapindaceae | Harpullia rhyticarpa |  |  |
|  | Toechima erythrocarpum |  |  |
| Sapotaceae | Palaquium galactoxylum |  |  |
|  | Pouteria xerocarpa |  |  |
| Sterculiaceae | Argyrodendron peralatum |  |  |
| Vitaceae | Leea indica |  |  |
