# Supplementary material for: Broad Anatomical Variation within a Narrow Wood Density Range—A Study of Twig Wood across 69 Australian Angiosperms
Source: PLoS One. 2015 Apr 23;10(4):e0124892. doi: 10.1371/journal.pone.0124892 (PMC4408027; doi:10.1371/journal.pone.0124892)

**Density of cell wall material**

Density of *swollen* cell wall has been shown to be around 1 g cm^-3^ (as oppose to *dry* cell wall material with 1.5 g cm^-3^) and relatively consistent across species ([1], and literature cited therein). However, in our dataset, the calculated swollen cell wall density varied from 1.09 to 2.66 g cm^-3^ (stack bar graph in Fig. 1). There are several reasons for this discrepancy as discussed below.

Swollen cell wall material density can be calculated as overall wood density divided by total cell wall fraction [1]. And the overall wood density is measured as the ratio of dry wood mass to green or soaked volume (as it is done in many ecological studies, [e.g. 2,3–5]) and cell wall fraction is calculated on swollen material (e.g. soaked material). Here, we did not use total cell wall fraction but fibre wall fraction plus vessel wall fraction (called here ‘wall_F+V_ fraction’), as they were the only wall fractions we had measures for (the remaining ones being parenchyma and conduits_15μm_ walls). So that cell wall material density was calculated as follows: overall wood density / wall_F+V_ fraction (Fig. 1, data in Supporting Information).

A first reason for the discrepancy might be that we did not use the total wall fraction. If we had, the calculated values of cell wall material would have been lower, and hence more in concordance with previous studies ([1], and literature cited therein). A second reason might be that our overall density measurements were overestimated for species, which contained substances such as for example starch or mucilage (starch density is approximately 1.5 g cm^-3^, [6,7]. If those substances had not contributed to the overall density, this density would have been lower, and the estimated density of cell wall material would similarly have been lower. A third reason is related to the method of cell wall fraction measurements. [1] have shown a discrepancy in wall and lumen fraction estimations from blocks of wood *vs*. from cross-sections. This is because a cut block of wood and a cross-section undergo different levels of deformation (swelling or shrinkage of walls and lumens) during the preparation process. We did not estimate fractions from a twig but only from a cross-section and have no reference point to estimate the error. Nevertheless, for example, in [1] the wall proportion estimated from a wet section varied from 4.4% lower to 9.7% higher relative to the fraction estimated from a surface of a wet block (across five angiosperms and two gymnosperms). Moreover, the authors have indicated that the error was bigger for lower density species. All those reasons were likely to have contributed to the relatively wide variation in the estimated density of swollen cell wall material in this study.

It is also worth noting, that density of cell wall material has frequently been cited to be c. 1.5 g cm^-3^ [8–11]. This value is correct for *dry* cell wall density, but not for *swollen* cell wall density (1 g cm^-3^, discussed above). In certain studies, it may be crucial to use the appropriate cell wall material density.

**References**

1. Kellogg R, Wangaard F (1969) Variation in the cell-wall density of wood. Wood Fiber Sci 1: 180–204.

2. Martínez-Cabrera HI, Jones CS, Espino S, Schenk HJ (2009) Wood anatomy and wood density in shrubs: responses to varying aridity along transcontinental transects. Am J Bot 96: 1388–1398. doi:10.3732/ajb.0800237.

3. Poorter L, McDonald I, Alarcón A, Fichtler E, Licona J, et al. (2010) The importance of wood traits and hydraulic conductance for the performance and life history strategies of 42 rainforest tree species. New Phytol 185: 481–492. doi:10.1111/j.1469-8137.2009.03092.x.

4. Fan Z-X, Zhang S-B, Hao G-Y, Ferry Slik J w., Cao K-F (2012) Hydraulic conductivity traits predict growth rates and adult stature of 40 Asian tropical tree species better than wood density. J Ecol 100: 732–741. doi:10.1111/j.1365-2745.2011.01939.x.

5. Fortunel C, Ruelle J, Beauchêne J, Fine PVA, Baraloto C (2014) Wood specific gravity and anatomy of branches and roots in 113 Amazonian rainforest tree species across environmental gradients. New Phytol 202: 79–94. doi:10.1111/nph.12632.

6. Gordon R (1987) A retaliatory role for algal projectiles, with implications for the mechanochemistry of diatom gliding motility. J Theor Biol 126: 419–436. doi:10.1016/S0022-5193(87)80149-2.

7. Rodriguez-Perez MA, Simoes RD, Constantino CJL, de Saja JA (2011) Structure and physical properties of EVA/starch precursor materials for foaming applications. J Appl Polym Sci 121: 2324–2330. doi:10.1002/app.33946.

8. Van Gelder HA, Poorter L, Sterck FJ (2006) Wood mechanics, allometry, and life-history variation in a tropical rain forest tree community. New Phytol 171: 367–378. doi:10.1111/j.1469-8137.2006.01757.x.

9. Chave J, Coomes D, Jansen S, Lewis SL, Swenson NG, et al. (2009) Towards a worldwide wood economics spectrum. Ecol Lett 12: 351–366. doi:10.1111/j.1461-0248.2009.01285.x.

10. McCulloh KA, Johnson DM, Meinzer FC, Voelker SL, Lachenbruch B, et al. (2012) Hydraulic architecture of two species differing in wood density: opposing strategies in co-occurring tropical pioneer trees. Plant Cell Environ 35: 116–125. doi:10.1111/j.1365-3040.2011.02421.x.

11. Swenson NG (2012) The functional ecology and diversity of tropical tree assemblages through space and time: from local to regional and from traits to transcriptomes. ISRN For 2012: 1–16. doi:10.5402/2012/743617.

**Figure 1. Bar graph representing estimated density of swollen cell wall material across 69 studied species.** Each bar corresponds to one species and the bars are ordered from the lowest (left side) to the highest density (right side). Values calculated for three replicates per species (with five exceptions mentioned in ‘Materials and methods’ section. Whiskers indicate standard deviation. Red line corresponds to the density of 1 g cm^-3^, which is approximately the density of swollen cell wall material measured by Kellogg and Wangaard (1969). * species from the tropical woodland, ** species from the temperate forest.


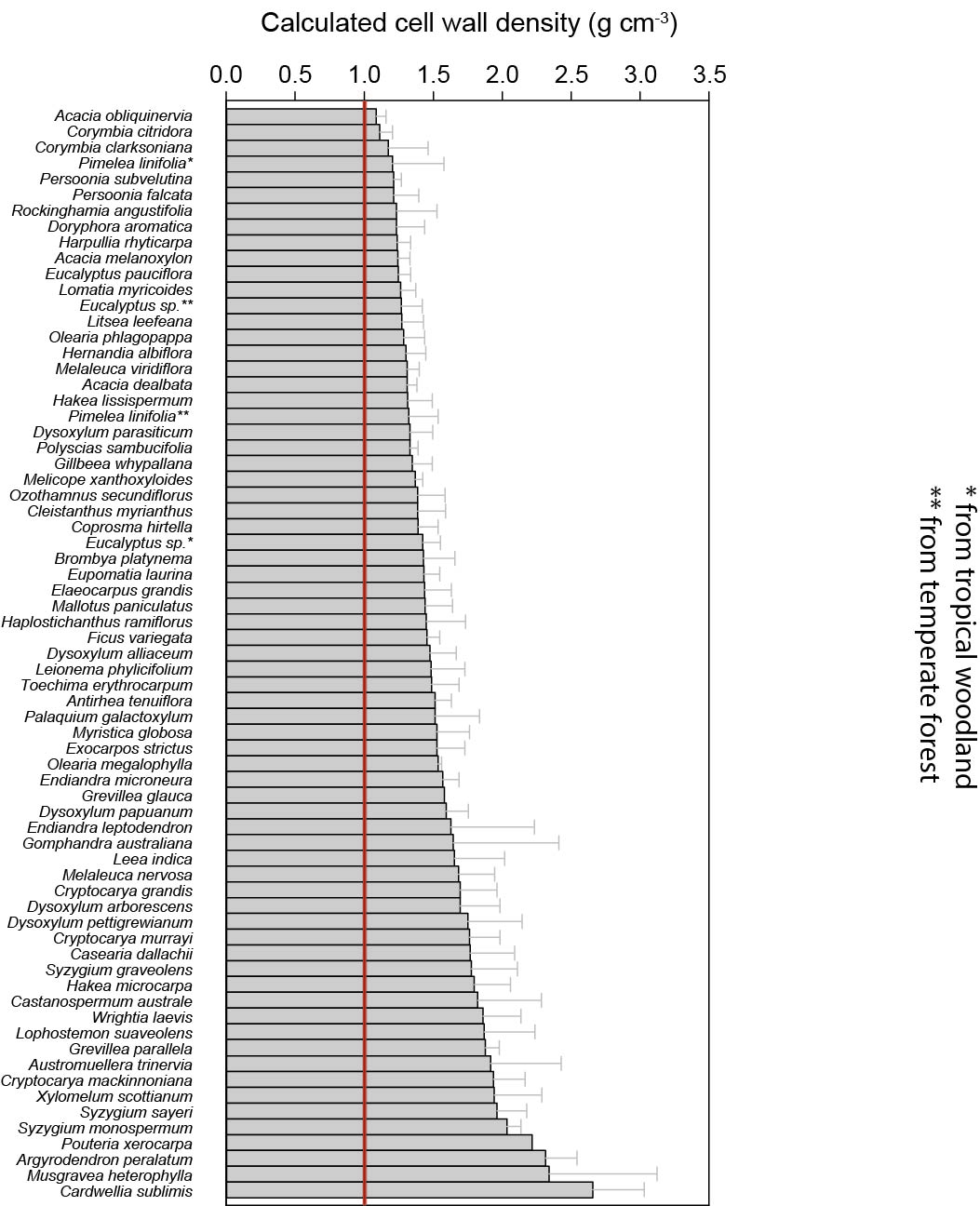

Supplement: S1 Text — (DOCX) [file pone.0124892.s007.docx]
