# Supplementary material for: Broad Anatomical Variation within a Narrow Wood Density Range—A Study of Twig Wood across 69 Australian Angiosperms
Source: PLoS One. 2015 Apr 23;10(4):e0124892. doi: 10.1371/journal.pone.0124892 (PMC4408027; doi:10.1371/journal.pone.0124892)

Site comparisons

The sampling from three diverse climates and vegetation types broadened significantly the number and diversity of species studied here (see Table 1 in the main article for site details). However, due to the lack of site replication within a given climate and non-random species selection, it is not possible to draw reliable conclusions about climate/vegetation type differences and only tentative observations and interpretations can be made.

To compare the sites one-way ANOVA was used (Holm-Šidák post-hoc test) for normally distributed traits with equal variances. For the traits with not normal distributions or unequal variances, ANOVA on ranks was used (Kruskal-Wallis test, Dunn’s post-hoc test). The alpha level of performed tests was 0.05. Table 1 summarizes site means, medians, and ANOVA results with significance values.

There were significant differences between sites in parenchyma traits. Total parenchyma was most abundant in the tropical rainforest (fraction median = 0.39, Fig. 1A), and this mainly resulted from ray fraction being highest in that site (median = 0.23 *vs*. 0.16 in the tropical woodland and 0.15 in the temperate forest, Fig. 1B). Average axial parenchyma fraction was similar across the three sites (0.15 in the tropical rainforest, 0.14 in the tropical woodland and 0.10 in the temperate forest, Fig. 1C). Parenchyma traits did not differ significantly between the tropical woodland and the temperate forest.

Average vessel area was significantly smaller at the cool temperate forest site then at the two tropical sites. Between those two tropical sites, which have similar temperature but markedly different rainfall, vessel area did not differ significantly (Fig. 1D). Hydraulically weighted diameter mirrored the patterns of vessel area, but with even more pronounced site differences. Conduits_15μm_ are a potentially conductive tissue composed of very narrow cells (maximum lumen diameter smaller than 15μm). Conduits_15μm_ occurred in only 0.05% of species (2 of 41) from the rainforest (warm-wet site), but in 91% of species (10 of 11) from the tropical woodland (warm-dry site) and 82% (14 of 17) in the temperate forest (cold-wet site). In general, species from the rainforest site had large vessels with none or few conduits_15μm_, species from the woodland site had large vessels with abundant conduits_15μm_, and species from the temperate forest site had small vessels with abundant conduits_15μm_ (Fig. 1E).

At the tropical woodland and the temperate forest sites only *Pimelea linifolia* had density < 0.5 g cm^-3^. In comparison, more than half of the species (23 of 41) at the tropical rainforest site had density < 0.5 g cm^-3^. Not surprisingly, mean wood density was significantly lower in the rainforest than in the two other sites, among which there was no significant difference (Fig. 1F). In interpreting these results, it needs to be remembered that these species were not sampled at random from all species present, but selectively from the medium and low range of wood density (and, further, that there was no replication within site-types, so these results are best thought of as indicative only). Because density tends to vary with fibre wall and lumen fractions [1–6], differences in those tissues across the three sites could be expected to mirror the differences in density. This was the case for fibre wall fraction but not for fibre lumen, indicating that lower density in the rainforest was mainly driven by fibre wall fraction, and other tissue component could have possibly secondary effects (e.g. fibre lumen or parenchyma or conduits_15μm_ wall fractions).

The stiffest wood material (highest MOE) was observed in the temperate woodland, while the two tropical sites did not differ significantly. Leaf area to sapwood area ratio was larger in the rainforest site than in the two other sites, which did not differ significantly from one another in this respect.

**Site comparisons discussion**

Ray and axial parenchyma fractions had different patterns of variation across the sites. The absolute spread of ray fraction was similar across sites, but in the tropical rainforest it was shifted towards higher values (Fig. 1B). In contrast, the spread of axial parenchyma fractions varied across sites. The widest spread was at the tropical rainforest, and it encompassed the spread found at the tropical woodland and the temperate forest (Fig. 1C). Across 81 species of *Ilex* (mainly mature stems), it has been observed that axial parenchyma is more abundant in the tropics than in the temperate regions ([7]; although we note that the method of assessing parenchyma abundance or the statistical analysis used were not clear in that publication). In addition, a global wood anatomy dataset indicates that the incidence of species with more abundant axial parenchyma is higher in the tropics [8]. In that study parenchyma abundance was not measured directly but rather abundance categories were used and it is difficult to draw comparisons. Tentatively we can say that in twigs, similar to the mature stems analysed by [8], it is rather the incidence of species with abundant axial parenchyma that increases towards the tropics, while species with little axial parenchyma continue to be present. An increased incidence of species with abundant parenchyma towards the tropics could possibly also be consistent with a positive relationship between axial parenchyma and temperature observed across 61 woody shrubs of North and South Americas (*r* = 0.33, *P* < 0.05, [4]).

Our second question was concerned with parenchyma trends across the precipitation gradient and we hypothesized higher parenchyma fractions at the drier, more seasonal site. In fact we did not find any difference in axial parenchyma fractions between the tropical rainforest and the tropical woodland but rays occupied larger fractions in the tropical rainforest (the wet site) than in the tropical woodland (the dry site). In a dataset of 61 species spanning across South and North America, axial parenchyma tended to be more abundant in drier sites, while ray fraction was more abundant in wetter sites, but the correlations were weak (*r* = -0.35, *r* = 0.30, both *P* < 0.05; [4]). Across 111 tree species from tropical climates in Africa and South America, total parenchyma fraction tended weakly to decrease with precipitation (*r* = - 0.35, *P* < 0.05, mean annual rainfall varied from *c*. 450 mm to 4200 mm), and weakly to increase with the length of stress period (*r* = 0.24, *P* < 0.01, [9]), while no correlation was found with precipitation in a transcontinental study across 61 species (precipitation range 100-1850mm; [4]).

Within the tropics, we expected smaller vessels in the dry site (the tropical woodland) than in the wet site (the tropical rainforest). The dry site had four times lower precipitation during the wettest month than the wet site, and also a longer dry season (Table 1 in the main article). These conditions would be expected to lead to lower stem water potentials, carrying higher risk of drought-induced embolism. Small vessels are less prone to drought-induced embolism [10–12]. Surprisingly though, there was no difference in vessel area medians in our study, although variation between species was wider at the wet site, and included a few species with large vessel areas not found in the dry site (Fig. 1D). Possibly the vessel size traits at the dry site can be thought of as adapted to take advantage of the wet season, even though it is short. Strategies other than vessel size may play a protective role during the dry season [13,14]. These could include fine-scale modifications of intervessel pitting [15,16]. [15] found the highest incidence of species with vestured pits in deserts and in tropical seasonal woodlands. Vestured pits have been suggested to participate in embolism resistance [17–19]. Another relevant strategy might be the fraction of very small conduits (conduits narrower than 15 μm here called ‘conduits_15μm_’, which could be small vessels or tracheids). These occurred in over 90% of species from the dry site and only in 5% of species from the wet. These conduits can potentially safely transport or store water [20] and have been suggested as playing a significant role in water stressed regions [21–24].

The fraction of conduits_15μm_ was also relatively high in the temperate forest where temperatures below 0°C occur in winter. These climatic conditions are prone to freeze-thaw embolism. It has been shown that smaller vessels are more resistant to this type of embolism [25,26]. Indeed, in our study species from the temperate forest had significantly smaller vessels than in the tropical sites, as well as a larger fraction of conduits_15μm_. These both would be expected to reduce the risk of freeze-thaw embolism.

References

1. Fujiwara S, Sameshima K, Kuroda K, Takamura N (1991) Anatomy and properties of Japanese hardwoods. I. Variation of fibre dimensions and tissue proportions and their relation to basic density. IAWA Bull Ns 12: 419–424.

2. Jacobsen AL, Agenbag L, Esler KJ, Pratt RB, Ewers FW, et al. (2007) Xylem density, biomechanics and anatomical traits correlate with water stress in 17 evergreen shrub species of the Mediterranean‐type climate region of South Africa. J Ecol 95: 171–183. doi:10.1111/j.1365-2745.2006.01186.x.

3. Rana R, Langenfeld-Heyser R, Finkeldey R, Polle A (2009) Functional anatomy of five endangered tropical timber wood species of the family Dipterocarpaceae. Trees - Struct Funct 23: 521–529. doi:10.1007/s00468-008-0298-4.

4. Martínez-Cabrera HI, Jones CS, Espino S, Schenk HJ (2009) Wood anatomy and wood density in shrubs: responses to varying aridity along transcontinental transects. Am J Bot 96: 1388–1398. doi:10.3732/ajb.0800237.

5. Ziemińska K, Butler DW, Gleason SM, Wright IJ, Westoby M (2013) Fibre wall and lumen fractions drive wood density variation across 24 Australian angiosperms. AoB PLANTS 5. Available: http://aobpla.oxfordjournals.org/content/5/plt046.full. Accessed 3 January 2014.

6. Fortunel C, Ruelle J, Beauchêne J, Fine PVA, Baraloto C (2014) Wood specific gravity and anatomy of branches and roots in 113 Amazonian rainforest tree species across environmental gradients. New Phytol 202: 79–94. doi:10.1111/nph.12632.

7. Baas P (1973) The wood anatomical range in Ilex (Aquifoliaceae) and its ecological and phylogenetic significance. Blumea 21: 193–258.

8. Wheeler EA, Baas P, Rodgers S (2007) Variations in dicot wood anatomy: a global analysis based on the InsideWood database. IAWA J 28: 229–258.

9. Fichtler E, Worbes M (2012) Wood anatomical variables in tropical trees and their relation to site conditions and individual tree morphology. IAWA J 33: 119–140.

10. Lo Gullo MA, Salleo S (1993) Different vulnerabilities of Quercus ilex L. to freeze- and summer drought-induced xylem embolism: an ecological interpretation. Plant Cell Environ 16: 511–519. doi:10.1111/j.1365-3040.1993.tb00898.x.

11. Hargrave KR, Kolb KJ, Ewers FW, Davis S d. (1994) Conduit diameter and drought-induced embolism in Salvia mellifera Greene (Labiatae). New Phytol 126: 695–705. doi:10.1111/j.1469-8137.1994.tb02964.x.

12. Wheeler JK, Sperry JS, Hacke UG, Hoang N (2005) Inter-vessel pitting and cavitation in woody Rosaceae and other vesselled plants: a basis for a safety versus efficiency trade-off in xylem transport. Plant Cell Environ 28: 800–812. doi:10.1111/j.1365-3040.2005.01330.x.

13. Crivellaro A, McCulloh K, Jones FA, Lachenbruch B (2012) Anatomy and mechanical and hydraulic needs of woody climbers contrasted with subshrubs on the island of Cyprus. Iawa J 33: 355–373.

14. Gleason SM, Butler DW, Waryszak P (2013) Shifts in leaf and stem hydraulic traits across aridity gradients in eastern Australia. Int J Plant Sci 174: 1292–1301. doi:10.1086/673239.

15. Jansen S, Baas P, Gasson P, Lens F, Smets E (2004) Variation in xylem structure from tropics to tundra: Evidence from vestured pits. Proc Natl Acad Sci U S A 101: 8833–8837. doi:10.1073/pnas.0402621101.

16. Choat B, Cobb AR, Jansen S (2008) Structure and function of bordered pits: new discoveries and impacts on whole-plant hydraulic function. New Phytol 177: 608–626. doi:10.1111/j.1469-8137.2007.02317.x.

17. Zweypfenning RCVJ (1978) A hypothesis on the function of vestured pits. IAWA Bull 1: 13–15.

18. Jansen S, Baas P, Gasson P, Smets E (2003) Vestured pits: do they promote safer water transport? Int J Plant Sci 164: 405–413. doi:10.1086/374369.

19. Choat B, Jansen S, Zwieniecki MA, Smets E, Holbrook NM (2004) Changes in pit membrane porosity due to deflection and stretching: the role of vestured pits. J Exp Bot 55: 1569–1575. doi:10.1093/jxb/erh173.

20. Sano Y, Morris H, Shimada H, Craene LPRD, Jansen S (2011) Anatomical features associated with water transport in imperforate tracheary elements of vessel-bearing angiosperms. Ann Bot 107: 953–964. doi:10.1093/aob/mcr042.

21. Carlquist S (1984) Vessel grouping in dicotyledon wood: significance and relationship to imperforate tracheary elements. Aliso 10: 505–525.

22. Carlquist S (1985) Vasicentric tracheids as a drought survival mechanism in the woody flora. Aliso 11: 37–68.

23. Carlquist S (2012) How wood evolves: a new synthesis. Botany 90: 901–940. doi:10.1139/b2012-048.

24. Carlquist S, Hoekman DA (1985) Ecological wood anatomy of the woody Southern Californian flora. IAWA Bull Ns 6: 319–347.

25. Davis SD, Sperry JS, Hacke UG (1999) The relationship between xylem conduit diameter and cavitation caused by freezing. Am J Bot 86: 1367–1372.

26. Pittermann J, Sperry J (2003) Tracheid diameter is the key trait determining the extent of freezing-induced embolism in conifers. Tree Physiol 23: 907–914. doi:10.1093/treephys/23.13.907.

**Table 1. Site comparisons.**

| Trait | Units | Tropical rainforest (R) | Tropical woodland (W) | Temperate forest (TF) | ANOVA | Post-hoc test | | |
| --- | --- | --- | --- | --- | --- | --- | --- | --- |
|  |  |  |  |  |  | R vs. W | R vs. TF | W vs. TF |
|  |  | AV or *M* | AV or *M* | AV or *M* | F or *H* | t or *Q* | t or *Q* | t or *Q* |
| Axial parenchyma fraction | unitless | 0.15 | 0.14 | 0.10 | 3.33* | 0.64^ns^ | 2.58* | 1.36^ns^ |
| Ray parenchyma fraction | " | *0.23* | *0.16* | *0.15* | *17.22**** | *2.70** | *3.72** | *0.40* ^ns^ |
| Total parenchyma fraction (axial + ray) | " | *0.39* | *0.25* | *0.24* | *20.50**** | *2.44** | *4.30** | *1.07* ^ns^ |
| Fibre lumen fraction | " | 0.14 | 0.11 | 0.14 | 0.73 ^ns^ | na | na | na |
| Fibre wall fraction | " | 0.28 | 0.35 | 0.38 | 18.19*** | 3.38** | 5.68*** | 1.26 ^ns^ |
| Total fibre fraction (lumen + wall) | " | 0.41 | 0.46 | 0.52 | 7.10** | 1.42^ns^ | 3.73*** | 1.54 ^ns^ |
| Vessel lumen fraction | " | 0.14 | 0.15 | 0.11 | 5.17** | 1.13^ns^ | 2.63* | 2.96* |
| Vessel wall fraction | " | *0.04* | *0.04* | *0.04* | *0.051* ^ns^ | *na* | *na* | *na* |
| Total vessel fraction (lumen + wall) | " | 0.18 | 0.20 | 0.16 | 2.45 ^ns^ | na | na | na |
| Fraction of conduits with maximum lumen diameter smaller than 15 μm | " | *0.00* | *0.05* | *0.05* | *46.15**** | *4.21** | *5.07** | *0.08* ^ns^ |
| Wall fraction (fibre wall + vessel wall) | " | 0.32 | 0.40 | 0.43 | 19.4*** | 3.42** | 5.89*** | 1.39^ns^ |
| Vessel area | mm^2^ | *0.0016* | *0.0015* | *0.0004* | *22.15**** | *0.72^ns^* | *4.68** | *2.86** |
| Vessel number per area | mm^-2^ | *92* | *104* | *266* | *12.44*** | *1.21^ns^* | *3.61** | *1.56* ^ns^ |
| Vessel area to number ratio | mm^4^ | *0.000019* | *0.000012* | *0.000001* | *14.83**** | *1.15^ns^* | *3.85** | *1.86* ^ns^ |
| Hydraulically weighted diameter | mm | 55 | 58 | 33 | 21.10*** | 0.69^ns^ | 6.08*** | 5.14*** |
| Pith area | mm^2^ | *4.13* | *0.69* | *0.77* | *35.56**** | *4.33** | *5.05** | *0.03* ^ns^ |
| Wood density | g cm^-3^ | 0.50 | 0.56 | 0.57 | 11.67*** | 3.06** | 4.38*** | 0.58 ^ns^ |
| Height | m | *19.0* | *3.6* | *2.0* | *38.31**** | *3.94** | *5.61** | *0.72* ^ns^ |
| Maximum height | m | *20* | *10* | *3.5* | *17.45**** | *1.65* ^ns^ | *4.12** | *1.62* ^ns^ |
| Modulus of elasticity | MPa | *4455* | *3380* | *7964* | *21.95**** | *1.26* ^ns^ | *4.06** | *4.13** |
| Leaf area / sapwood area | cm^2^ cm^-2^ | *9398* | *5369* | *2357* | *42.29**** | *3.04** | *6.33** | *2.05* ^ns^ |

Notes: AV – average, *M* – median, R – tropical rainforest, W – tropical woodland, TF – temperate forest, F – ANOVA statistic, H – ANOVA on ranks statistic (Kruskal-Wallis), t – post-hoc statistic (Holm-Šidák method), Q – post-hoc test statistic (Dunn’s method), ^ns^ - not significant, * - *P* ≤ 0.05, ** - P ≤ 0.01, *** - P ≤ 0.001. Results from ANOVA are in roman font, results from ANOVA on ranks (Kruskal-Wallis) are in italic font.

**Figure 1. Box plots showing differences in anatomical traits and wood density between sites (and vegetation types): tropical rainforest (warm and wet site), tropical woodland (warm and dry site), and temperate forest (cool and wet site).** The black line inside the grey box is a median. The box top and bottom boundaries indicate upper and lower quartile and the whiskers are highest and lowest values excluding outliers. Circles are outliers and each circle corresponds to an individual species.


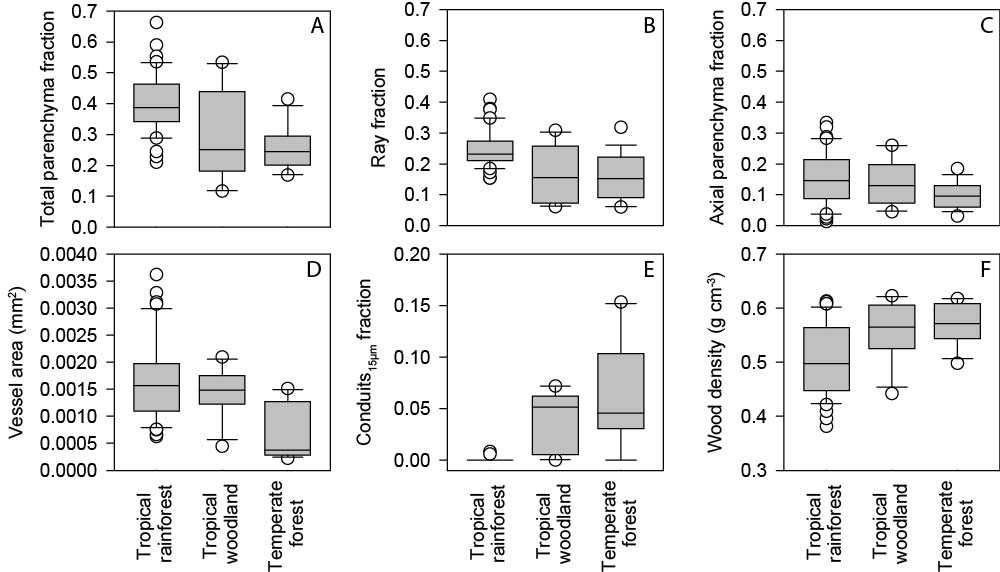

Supplement: S2 Text — (DOCX) [file pone.0124892.s008.docx]
